# Comparative efficacy of gait training for balance outcomes in patients with stroke: A systematic review and network meta-analysis

**DOI:** 10.3389/fneur.2023.1093779

**Published:** 2023-04-03

**Authors:** Tianyi Lyu, Kang Yan, Jiaxuan Lyu, Xirui Zhao, Ruoshui Wang, Chaoyang Zhang, Meng Liu, Chao Xiong, Chengjiang Liu, Yulong Wei

**Affiliations:** ^1^School of Acupuncture-Moxibustion and Tuina, Beijing University of Chinese Medicine, Beijing, China; ^2^L3 & Maintenance Solutions, SUSE Software (Beijing) Co., Ltd., Beijing, China; ^3^Department of General Medicine, Affiliated Anqing First People’s Hospital of Anhui Medical University, HeFei, Anhui, China

**Keywords:** stroke, gait training, network meta-analysis, randomized controlled trials, balance outcomes

## Abstract

**Background:**

Growing evidence suggests that gait training can improve stroke patients’ balance outcomes. However, it remains unclear which type of gait training is more effective in improving certain types of balance outcomes in patients with stroke. Thus, this network meta-analysis (NMA) included six types of gait training (treadmill, body-weight-supported treadmill, virtual reality gait training, robotic-assisted gait training, overground walking training, and conventional gait training) and four types of balance outcomes (static steady-state balance, dynamic steady-state balance, proactive balance, and balance test batteries), aiming to compare the efficacy of different gait training on specific types of balance outcomes in stroke patients and determine the most effective gait training.

**Method:**

We searched PubMed, Embase, Medline, Web of Science, and Cochrane Library databases from inception until 25 April 2022. Randomized controlled trials (RCTs) of gait training for the treatment of balance outcomes after stroke were included. RoB2 was used to assess the risk of bias in the included studies. Frequentist random-effects network meta-analysis (NMA) was used to evaluate the effect of gait training on four categories of balance outcomes.

**Result:**

A total of 61 RCTs from 2,551 citations, encompassing 2,328 stroke patients, were included in this study. Pooled results showed that body-weight-support treadmill (SMD = 0.30, 95% CI [0.01, 0.58]) and treadmill (SMD = 0.25, 95% CI [0.00, 0.49]) could improve the dynamic steady-state balance. Virtual reality gait training (SMD = 0.41, 95% CI [0.10, 0.71]) and body-weight-supported treadmill (SMD = 0.41, 95% CI [0.02, 0.80]) demonstrated better effects in improving balance test batteries. However, none of included gait training showed a significant effect on static steady-state balance and proactive balance.

**Conclusion:**

Gait training is an effective treatment for improving stroke patients’ dynamic steady-state balance and balance test batteries. However, gait training had no significant effect on static steady-state balance and proactive balance. To achieve maximum efficacy, clinicians should consider this evidence when recommending rehabilitation training to stroke patients. Considering body-weight-supported treadmill is not common for chronic stroke patients in clinical practice, the treadmill is recommended for those who want to improve dynamic steady-state balance, and virtual reality gait training is recommended for those who want to improve balance test batteries.

**Limitation:**

Missing evidence in relation to some types of gait training is supposed to be taken into consideration. Moreover, we fail to assess reactive balance in this NMA since few included trials reported this outcome.

**Systematic Review Registration:**

PROSPERO, identifier CRD42022349965.

## Introduction

Recently, stroke is the leading cause of death in China and the second leading cause of death worldwide (1, 2). Despite the fact that stroke mortality, prevalence, and incidence have decreased in the past 20 years, its prevalence is increasing in young individuals (3). Thus, the significance of stroke rehabilitation has grown. Balance disorders, one of the most common symptoms after a stroke, can affect patients’ physiological and social functions (4, 5). Therefore, balance disorders place a heavy burden on both individuals and society.

Balance is one of the main functional goals of postural control and involves the coordination of movement strategies to stabilize the center of body mass during self-initiated and externally triggered stability perturbations (6). Balance disorders account for a series of gait-related disabilities, including problems with transferring, maintaining body posture, and locomotion (7, 8). Therefore, balance is an important component of gait to stabilize one’s body during mobility. Meanwhile, growing evidence suggests that gait training can improve balance outcomes (9, 10).

Gait training refers to specific types of physical therapies that help individuals strengthen and improve their walking capacity (11). Treadmill, body-weight-supported treadmill, robot-assisted gait training, virtual reality gait training, conventional gait training, and overground walking training are common types of gait training that have the potential to improve balance capacity (details in Table 1). Several studies have suggested that the aforementioned gait training could counteract the balance dysfunction caused by various diseases, such as stroke and Parkinson’s disease (9, 10). However, the effects of gait training on balance rehabilitation after stroke have been inconclusive. It remains unclear which type of gait training is the most effective. Canadian Guideline revealed that gait training (e.g., body-weight-supported treadmill) might improve dynamic balance in the subacute phase after stroke (12). A recent meta-analysis suggested that overground walking training and robotic-assisted gait training showed no significant effect on balance outcomes while treadmill showed a significant effect on balance outcomes (13). Another systematic review and meta-analysis reported that no significant balance gains were obtained from gait training (e.g., body-weight-supported treadmill and robot-assisted gait training) (14).

**Table 1:** Characteristics of included gait training.

Type	Abbreviation	Description
Treadmill	TT	*gait training requires participants to walk on motorized treadmills without any other device assisted*
Body weight-supported treadmill	BWS-TT	*gait training uses a harness to provide partial body weight support in conjunction with a motorized treadmill*
Robot-assisted gait training	RA-GT	*gait training with robotic-orthosis attached to patients’ lower extremities, allowing the guidance force during ambulation*
Virtual reality gait training	VR	*gait training with VR systems, providing realistic environments in which people can have real-time interaction with objects and events*
Conventional gait training	CGT	*gait training administered by physiotherapist according to individual walking capacity of the participants*
Overground walking training	OWT	*gait training requires participants to walk around an outdoor circuit*

According to Shumway-Cook and Woollacott (15), balance performance can be divided into four types, including dynamic steady-state balance, static steady-state balance, proactive balance, and reactive balance. In addition, there are only small-sized correlations between different types of balance performance (16). With reference to these findings, balance outcome measures are further subdivided into five types, including static steady-state balance, dynamic steady-state balance, proactive balance, reactive balance, and balance test batteries (17). The first four types of balance outcome measures correspond, one by one, to the four types of balance performance, and the fifth type of balance outcome measure (balance test batteries) is added to assess the overall balance performance (18, 19). This classification has been used in several types of research to assess balance status and changes in response to exercise (20, 21). Thus, investigating the effect of gait training in specific type of balance outcomes might provide more comprehensive evidence in this field.

As mentioned earlier, evaluating the effects of gait training in post-stroke patients is of particular importance. However, selecting the optimal gait training to improve specific balance outcomes poses a challenge to clinicians. Because many gait training methods have not been directly compared in clinical trials, typical pairwise meta-analysis cannot be performed on them. Even when direct comparisons are available, the evidence is inadequate to make any conclusions. Therefore, we performed an NMA to compare the effects of different types of gait training on each type of balance outcome, thus identifying the optimal gait training for stroke survivors.

## Materials and methods

### Search strategy and selection criteria

The NMA was followed by the PRISMA statement (22), and the review protocol has been registered on PROSPERO (CRD42022349965). We searched PubMed, Embase, Medline, Web of Science, and Cochrane Library databases from inception until 25 April 2022, and the search strategy is described in Supplementary Table S1.

Inclusion criteria were based on the participants, interventions, comparators, outcomes, and study design (PICOS) approach (23). To be eligible for inclusion, studies had to meet the following criteria: (1) Population: adult stroke survivors who were considered suitable for gait training by the studies’ authors. (2) Intervention: treadmill, body-weight-supported treadmill, virtual reality gait training, robotic-assisted gait training, overground walking training, and conventional gait training. (3) Comparison: usual care, sham intervention, or no exercise intervention. (4) Outcome: balance outcome measurements (static steady-state balance, dynamic steady-state balance, proactive balance, and balance test batteries). In addition, we only assessed four types of balance outcomes due to the scarcity of studies on reactive balance. (5) Study design: randomized controlled trials.

The exclusion criteria were as follows: (1) intervention group including more than two types of gait training; (2) the type of physical exercise being unclear; (3) not reporting sufficient data to calculate the effect size; and (4) conference abstract without a fully published article.

### Data extraction and processing

Two independent investigators (M-L and Y-X) searched databases for relevant articles and removed duplicate records, with discrepancies adjudicated by a third investigator (TY-L). After removing duplicate records, pairs of independent investigators (CY-Z and XR-Z) screened references and extracted study-level data, with discrepancies adjudicated by a third investigator (TY-L). We also extracted study characteristics (name of the first author, country of origin, year of publication, intervention groups, and the sample size), participant characteristics (mean age, gender trends, and duration of the disease), and physical exercise characteristics (type, duration, frequency, and time).

### Outcomes

The main outcome data (expressed as mean and standard deviation) for changes in balance outcomes (static steady-state balance, dynamic steady-state balance, proactive balance, and balance test batteries) were extracted from initiation to the end of treatment. Only one representative outcome variable was included in the analysis when studies reported several variables under one of these outcome categories. In terms of dynamic steady-state balance, the highest priority was given to the usual walking speed. As a proxy for static steady-state balance, a single right leg stance with eyes opened was used. A functional reach test was preferably selected as a proxy for proactive balance. Berg balance scale was used as the most prominent balance test battery (17). If another test was used in a study, we chose to only include in our quantitative analysis those that had the most comparable temporal/spatial structure to the ones mentioned.

### Assessment of heterogeneity and inconsistency

We assessed heterogeneity by reporting the *I*^2^ statistic, as described in Section 9.5.2 of the Cochrane Handbook for Systematic Reviews of Interventions. Using this section, we used the following interpretation of the *I*^2^ statistic: 0–40% might not be important; 30–60% may represent moderate heterogeneity; 50–90% may represent substantial heterogeneity; and 75–100% may represent considerable heterogeneity (24). We used global and local methods to test the inconsistency of the research results. For global inconsistency, we evaluated inconsistency statistically using the design-by-treatment test (25). We assessed local inconsistency by splitting network estimates into the contribution of direct and indirect evidence (node-splitting test) (26). All heterogeneity and inconsistency analyses were performed with the statistical software R version 3.2.2.

### Risk-of-bias assessment

Two independent investigators (CY-Z and XR-Z) assessed the risk of bias of the included RCTs by RoB 2 revised Cochrane risk-of-bias tool for randomized trials. The results were incorporated into the Confidence in Network Meta-Analysis (CINeMA) application to evaluate the credibility of each NMA’s findings. CINeMA grade confidence was divided into four categories: high, moderate, low, or very low (27).

### Sensitivity analysis

We assessed the sensitivity of our findings by repeating each NMA after excluding studies at an overall high risk of bias. However, because more than one-third of the studies’ sample sizes were smaller than 30, sensitivity analysis was not performed on sample size (28).

### Statistical analysis

Each type of balance outcome was drawn to the network graph. The random-effects frequentist NMA was fitted to assume a common random effect for all comparisons in the network. Balance outcomes for each parameter and each treatment comparison were estimated as standard mean difference (SMD) with 95% confidence intervals (CIs). The control group was used as the reference group in all forest plots. Meanwhile, the league tables were created to display the relative degree of balance outcomes for all comparisons among gait training. P-scores were applied to rank gait training on the basis of balance outcomes. P-scores ranged from 0 to 1, with a higher P-score indicating a greater effect. In addition, we compared the comparison-adjusted funnel plot and Egger’s test to assess the risk of publication bias under specific circumstances, with Egger’s test suggesting publication bias when *p* < 0.05. Finally, the network plots and comparison-adjusted funnel plots were performed with StataSE version 16.0. Other network meta-analyses were performed with the statistical software R version 3.2.2.

## Results

### Literature search and characteristics of the included studies

**Figure 1:**
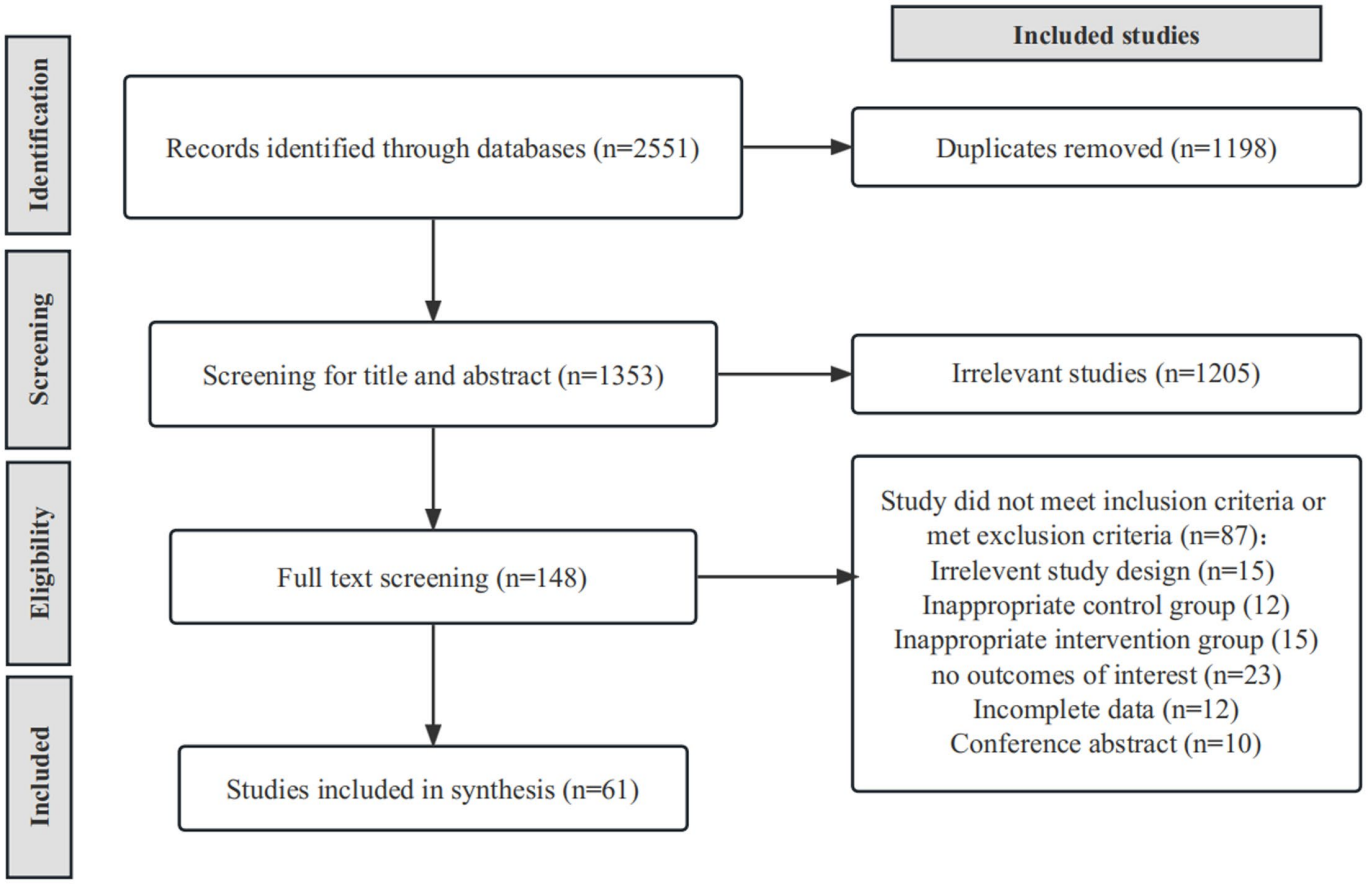
PRISMA flowchart illustrating the selection of studies included in our analysis.

After duplicate removal, we identified 1,353 relevant studies through a database searching strategy. After scanning titles and abstracts, we identified 148 articles. After full-text reading, we excluded 87 articles for reasons as follows: irrelevant study design (*n* = 15), inappropriate control group (*n* = 12), inappropriate intervention group (*n* = 15), no outcomes of interest (*n* = 23), incomplete data (*n* = 12), and conference abstract (*n* = 10). Finally, we identified 61 eligible RCTs (29–88) (Figure 1) containing 2,328 stroke patients, 1,157 (49.7%) of whom received gait training and 1,171 (50.3%) a control intervention. Overall, 1,318 (56.6%) of participants were men, and the mean age ranged from 44.2 to 74.8 years. The mean disease duration ranged from 19 days to 10.5 years. Supplementary Table S2 summarizes the characteristics of included RCTs.

### Intervention

There were six different types of gait training included in this NMA: (1) treadmill; (2) body-weight-supported treadmill; (3) virtual reality gait training; (4) robotic-assisted gait training; (5) overground walking training; and (6) conventional gait training.

Of 66 eligible studies, treadmill was assessed in 24 studies (29–32, 34, 35, 37, 45, 54, 57, 59, 62, 63, 65, 67–70, 72–75, 77, 78); body-weight-supported treadmill in 16 studies (32, 36, 38, 49, 59–62, 64, 66, 68, 71, 76, 83–85); conventional gait training in 9 studies (30, 38, 50, 51, 53, 56, 72, 82); overground walking training in 10 studies (36, 37, 60, 65, 67, 69–71, 74, 76); robotic-assisted gait training in 19 studies (31, 43, 46, 49–58, 66, 80–82, 87); and virtual reality gait training (33, 39–48, 73, 75, 77, 79, 86) in 16 studies.

### Risk of bias

**Figure 2:**
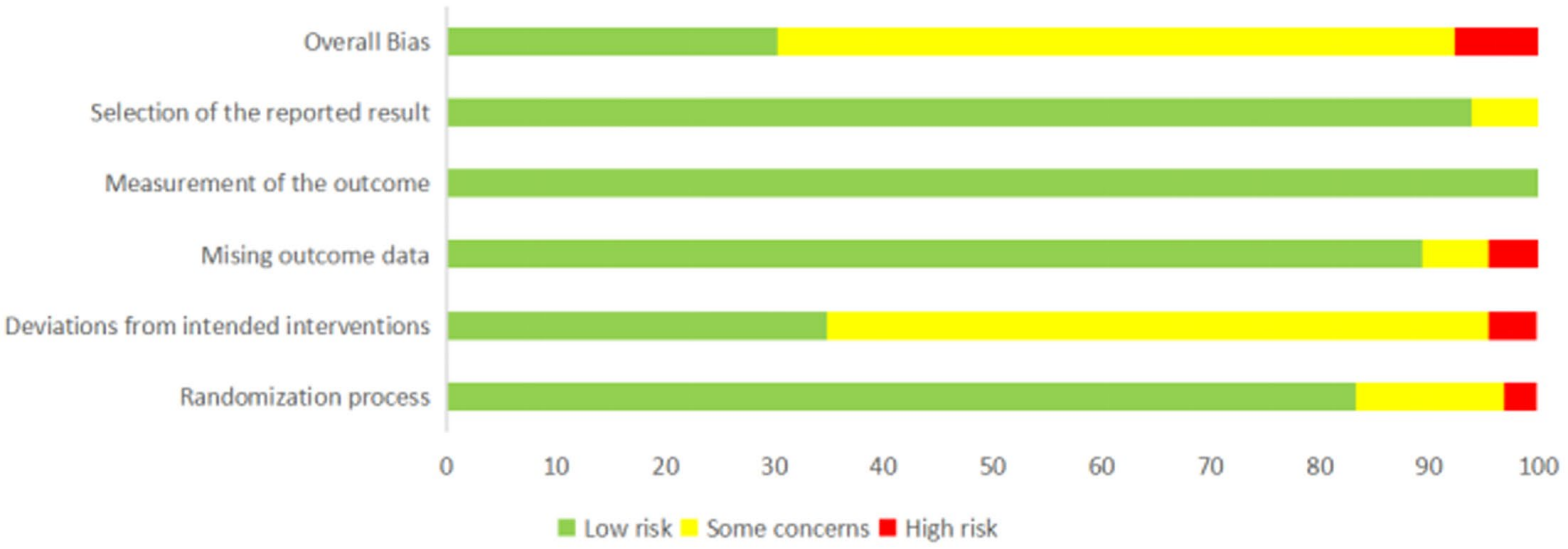
Risk of bias graph for each included study.

The RoB2 analysis showed that 18 (29.5%) studies had a low risk of bias, 38 (63.0%) had some concerns, and 5 (8.2%) had a high risk of bias (Figure 2; Supplementary Table S3). For the randomization process, nine (14.8%) studies had some concerns and two (3.3%) had a high risk of bias; for deviations from intended interventions, 37 (60.6%) studies had some concerns and 3 (4.9%) were at high risk of bias; for missing outcome data, four (6.6%) studies had some concerns and three (4.9%) had a high risk of bias; for measurement of the outcome, 61 (100%) studies had a low risk of bias; and for selection of the reported result, three (4.9%) studies had some concerns.

### Dynamic steady-state balance

**Figure 3:**
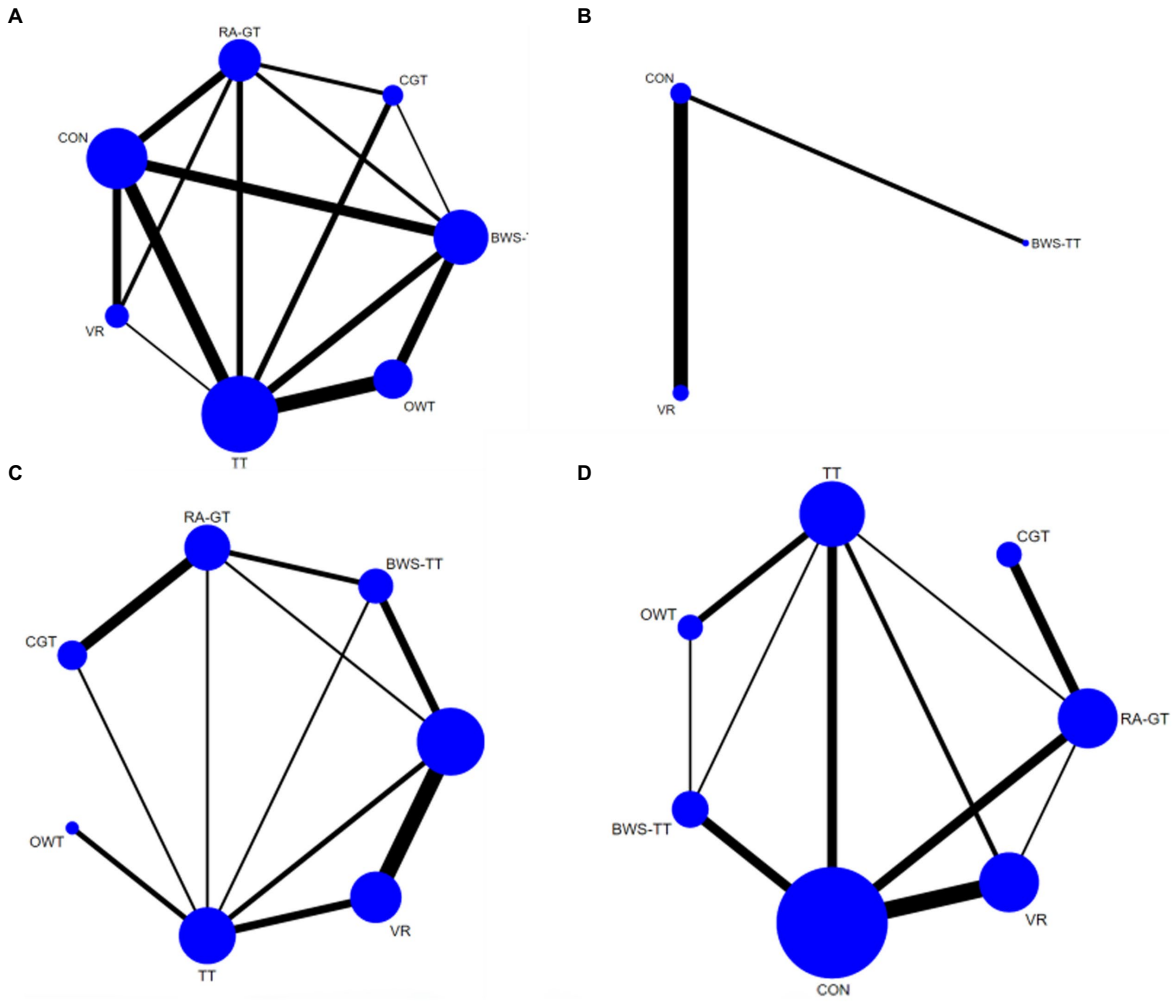
Network plots of the outcomes of the comparisons between gait training and controls in the NMA. **(A)** Dynamic steady-state balance; **(B)** Static steady-state balance; **(C)** Proactive balance; **(D)** Balance test batteries. TT, treadmill; BWS-TT, body-weight-supported treadmill training; RA-GT, robot-assisted gait training; VR, virtual-reality gait training; CGT, conventional gait training; OWT, overground walking training. Treatments with direct comparisons are linked with a line; the thickness of connecting lines corresponds to the number of trials evaluating the comparison; the size of the circle is proportional to the sample size.

**Figure 4:**
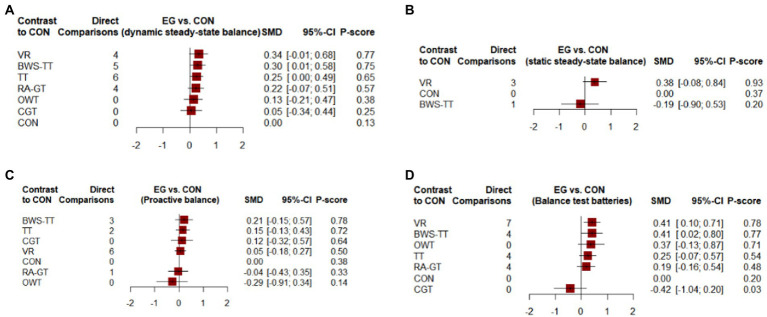
Forest plots of the efficiency of comparisons between gait training and controls. **(A)** Dynamic steady-state balance; **(B)** Static steady-state balance; **(C)** Proactive balance; **(D)** Balance test batteries. TT, treadmill; BWS-TT, body-weight-supported treadmill training; RA-GT, robot-assisted gait training; VR, virtual-reality gait training; CGT, conventional gait training; OWT, overground walking training. 95% CI ≥ 0 means intervention group is superior to control group significantly; P-scores were applied to rank gait training on the basis of balance outcome. P-scores ranged from 0 to 1, a higher P-core indicating a greater effect.

As shown in Figure 3A, the dynamic steady-state balance was available in 49 trials, comparing six types of gait training (517 patients) with control intervention (540 patients). Only body-weight-supported treadmill and treadmill appeared superior to control intervention significantly. Ranking on the basis of p-score identified virtual reality gait training as the best and conventional gait training as the worst (Figure 4A). No significant difference was found between gait training (Table 2). The *I*^2^ was 37.5%. Design-by-treatment test (*p* = 0.46, Supplementary Table S4A) and node-splitting test (0% comparison *p* < 0.05, Supplementary Table S5A) showed that no significant global and local inconsistency was observed. CINeMA grade confidence was low or very low in 14 (66.7%) comparisons (Supplementary Table S6A). Moreover, there was no publication bias in dynamic steady-state balance NMA (Egger’s test: *p* = 0.713, Supplementary Figure S1A).

**Table 2:** League table for dynamic steady-state balance estimate gait training according to their relative effects and 95% credibility intervals (95% CIs).

VR	NA	0.29 (−0.74; 1.32)	0.47 (−0.22; 1.16)	NA	NA	0.18 (−0.22; 0.59)
0.04 (−0.38; 0.46)	BWS-TT	0.34 (−0.12; 0.81)	0.30 (−0.34; 0.94)	−0.06 (−0.42; 0.31)	0.09 (−0.64; 0.81)	0.30 (−0.13; 0.73)
0.09 (−0.30; 0.48)	0.05 (−0.21; 0.31)	TT	−0.29 (−0.89; 0.32)	0.31 (−0.03; 0.65)	0.28 (−0.19; 0.75)	0.29 (−0.03; 0.61)
0.12 (−0.28; 0.51)	0.08 (−0.25; 0.40)	0.03 (−0.27; 0.33)	RA-GT	NA	0.14 (−0.47; 0.75)	0.31 (−0.11; 0.73)
0.20 (−0.25; 0.66)	0.16 (−0.12; 0.45)	0.12 (−0.16; 0.39)	0.09 (−0.29; 0.47)	OWT	NA	NA
0.29 (−0.20; 0.78)	0.25 (−0.14; 0.63)	0.20 (−0.15; 0.55)	0.17 (−0.22; 0.56)	0.08 (−0.34; 0.51)	CGT	NA
0.34 (−0.01; 0.68)	**0.30 (0.01; 0.58)**	**0.25 (0.00; 0.49)**	0.22 (−0.07; 0.51)	0.13 (−0.21; 0.47)	0.05 (−0.34; 0.44)	CON

Bold values mean significant difference. Blue means relative degree of direct comparison between gait training. Light red means relative degree of combined comparison between gait training. Deep red means gait training. TT, treadmill; BWS-TT, body-weight-supported treadmill training; RA-GT, robot-assisted gait training; VR, virtual reality gait training; CGT, conventional gait training; OWT, overground walking training; CON, control group.

### Static steady-state balance

As shown in Figure 3B, the static steady-state balance outcome was available in four trials, comparing two types of gait training (53 patients) with the control intervention (52 patients). Compared with the control intervention, none of the included gait training (body-weight-supported treadmill, virtual reality gait training) showed any significant effect. Ranking on the basis of p-score identified virtual reality gait training as the best and body-weight-supported treadmill as the worst (Figure 4B). No significant difference was found between gait training (Table 3). The *I^2^* was 0%. Due to the lack of direct comparison, we could not observe inconsistency between direct and indirect comparisons. CINeMA grade confidence was low or very low in three (100%) comparisons (Supplementary Table S5B). Moreover, we could not assess publication bias for the static steady-state balance due to the scarcity of studies.

**Table 3:** League table for static steady-state balance estimate gait training according to their relative effects and 95% credibility intervals (95% CIs).

BWS-TT	−0.19 (−0.90; 0.53)	.
−0.19 (−0.90; 0.53)	CON	−0.38 (−0.84; 0.08)
−0.57 (−1.42; 0.29)	−0.38 (−0.84; 0.08)	VR

Blue means relative degree of direct comparison between gait training. Light red means relative degree of combined comparison between gait training. Deep red means gait training. BWS-TT, body-weight-supported treadmill training; VR, virtual reality gait training; CON, control group.

### Proactive balance

As shown in Figure 3C, the proactive balance was available in 26 trials, comparing six types of gait training (437 patients) with control intervention (421 patients). Compared with the control intervention, none of the included gait training showed any significant effect. Ranking on the basis of p-score identified body-weight-supported treadmill as the best and overground walking training as the worst (Figure 4C). We did not find a significant difference in proactive balance between gait training according to the league table (Table 4). The *I*^2^ was 0%. Design-by-treatment test (*p* = 0.72, Supplementary Table S3C) and node-splitting test (0% comparison *p* < 0.05, Supplementary Table S4C) showed that no significant global and local inconsistency was observed. CINeMA grade confidence was low or very low in 14 (66.7%) comparisons. Moreover, we did not find publication bias in proactive balance NMA (Egger’s test: *p* = 0.909, Supplementary Figure S1B).

**Table 4:** League table for proactive balance estimate gait training according to their relative effects and 95% credibility intervals (95% CIs).

BWS-TT	NA	0.33 (−0.13; 0.79)	NA	0.16 (−0.36; 0.68)	−0.16 (−1.04; 0.72)	NA
0.08 (−0.39; 0.56)	CGT	NA	NA	0.21 (−0.17; 0.59)	−0.15 (−0.73; 0.42)	NA
0.21 (−0.15; 0.57)	0.12 (−0.32; 0.57)	CON	NA	0.34 (−0.39; 1.06)	−0.18 (−0.63; 0.27)	−0.04 (−0.29; 0.22)
0.49 (−0.19; 1.18)	0.41 (−0.29; 1.11)	0.29 (−0.34; 0.91)	OWT	NA	−0.44 (−1.00; 0.12)	NA
0.24 (−0.15; 0.64)	0.16 (−0.18; 0.49)	0.04 (−0.35; 0.43)	−0.25 (−0.93; 0.43)	RA-GT	0.35 (−0.57; 1.27)	NA
0.06 (−0.34; 0.45)	−0.03 (−0.45; 0.39)	−0.15 (−0.43; 0.13)	−0.44 (−1.00; 0.12)	−0.19 (−0.58; 0.20)	TT	0.08 (−0.27; 0.43)
0.16 (−0.25; 0.56)	0.07 (−0.39; 0.54)	−0.05 (−0.27; 0.18)	−0.33 (−0.96; 0.29)	−0.08 (−0.50; 0.34)	0.10 (−0.17; 0.38)	VR

Blue means relative degree of direct comparison between gait training. Light red means relative degree of combined comparison between gait training. Deep red means gait training. TT, treadmill; BWS-TT, body-weight-supported treadmill training; RA-GT, robot-assisted gait training; VR, virtual reality gait training; CGT, conventional gait training; OWT, overground walking training; CON, control group.

### Balance test batteries

As shown in Figure 3D, the balance test batteries were available in 32 trials, which compared six types of gait training (494 patients) with control intervention (485 patients). Balance test battery analysis showed that virtual reality gait training and body-weight-supported treadmill appeared superior to control intervention significantly. Ranking on the basis of p-score identified virtual reality gait training as the best and conventional gait training as the worst (Figure 4D). Virtual reality gait training, body-weight-supported treadmill, and robotic-assisted gait training appeared superior to conventional gait training (Table 5). The *I^2^* was 30%. Design-by-treatment test (*p* = 0.74, Supplementary Table S3D) and node-splitting test (0% comparison *p* < 0.05, Supplementary Table S4D) showed that no significant global and local inconsistency was observed. CINeMA grade confidence was low or very low in 17 (81.0%) comparisons. Moreover, there was no publication bias in balance test batteries NMA (Egger’s test: *p* = 0.503, Supplementary Figure S1C).

**Table 5:** League table for balance test batteries estimates gait training according to their relative effects and 95% credibility intervals (95% CIs).

VR	NA	NA	0.22 (−0.41; 0.86)	−0.05 (−0.92; 0.81)	**0.43 (0.07; 0.79)**	NA
−0.01 (−0.49; 0.47)	BWS-TT	−0.01 (−0.71; 0.70)	−0.09 (−0.97; 0.79)	NA	**0.51 (0.04; 0.98)**	NA
0.03 (−0.52; 0.59)	0.04 (−0.46; 0.54)	OWT	0.10 (−0.41; 0.61)	NA	NA	NA
0.16 (−0.23; 0.54)	0.16 (−0.26; 0.59)	0.12 (−0.32; 0.56)	TT	−0.59 (−1.66; 0.48)	0.31 (−0.11; 0.72)	NA
0.22 (−0.21; 0.64)	0.22 (−0.29; 0.74)	0.18 (−0.41; 0.77)	0.06 (−0.38; 0.50)	RA-GT	0.05 (−0.35; 0.44)	**0.61 (0.10; 1.13)**
**0.41 (0.10; 0.71)**	**0.41 (0.02; 0.80)**	0.37 (−0.13; 0.87)	0.25 (−0.07; 0.57)	0.19 (−0.16; 0.54)	CON	NA
**0.83 (0.16; 1.50)**	**0.84 (0.11; 1.56)**	0.80 (0.02; 1.58)	**0.67 (0.00; 1.35)**	**0.61 (0.10; 1.13)**	0.42 (−0.20; 1.04)	CGT

Bold values mean significant difference. Blue means relative degree of direct comparison between gait training. Light red means relative degree of combined comparison between gait training. Deep red means gait training. TT, treadmill; BWS-TT, body-weight-supported treadmill training; RA-GT, robot-assisted gait training; VR, virtual reality gait training; CGT, conventional gait training; OWT, overground walking training; CON, control group.

### Sensitivity analysis

The findings essentially remained the same in static steady-state balance and proactive balance sensitivity analyses (Supplementary Figures S2B,C). With regard to dynamic steady-state balance sensitivity analysis (Supplementary Figure S2A), the order of body-weight-supported treadmill and virtual reality gait training was reversed. However, virtual reality gait training did not have a significant effect on dynamic steady-state balance, thus, the removal of these studies did not have a significant effect on our primary results. With regard to balance test battery sensitivity analysis (Supplementary Figure S2D), the body-weight-supported treadmill achieved a higher p-score than virtual reality gait training but did not differ significantly from the control group. Hence, the primary findings from NMA remained unchanged after the sensitivity analysis. Assessments of heterogeneity in all sensitivity analyses were also similar.

## Discussion

Previous studies suggested that gait training showed its potential to improve the balance outcomes of stroke patients (10, 11). However, it remains unclear which type of gait training is more effective in improving specific balance outcomes. Therefore, this study aimed to summarize the related articles to compare the effects of different gait training on different types of balance outcomes in stroke patients. Moreover, our study has some strengths. First, a large number of adult stroke survivors (*n* = 2,328) were included. Second, to ensure a good level of evidence, we only included RCTs. Third, assessing balance gains in specific types also strengthened this study. Finally, no evidence of inconsistency, substantial heterogeneity, or publication bias was found in any NMA, and sensitivity analyses showed similar results to the overall findings.

To assess optimal gait training, this study performed an NMA to combine data from 61 RCTs including six different types of gait training of 2,328 stroke patients. Our main findings indicated that body-weight-supported treadmill and virtual reality gait training were the most promising gait training to improve dynamic steady-state balance and balance test batteries, respectively.

In terms of dynamic steady-state balance, body-weight-supported treadmill and treadmill showed significant efficacy when compared to control intervention, while the other five types of gait training did not. This result was consistent with the previous studies (89). Gait speed could be improved by repetitive mass motor task practice. When compared to conventional therapy, the body-weight-supported treadmill has the advantage of dynamic balance measured by gait speed because it provides more intensive, repetitive, and task-oriented training during the same time span (90). Moreover, body-weight-supported devices have a positive effect on spatial and temporal gait characteristics by improving body weight distribution between paretic and non-paretic parts (91). The treadmill was the second better gait training to improve dynamic steady-state balance. One possible explanation was that treadmill and body-weight-supported treadmill shared the same training process. Both of them involved repetitive mass motor task practice. In addition, patients’ characteristics, particularly stroke stage, should be taken into consideration when interpreting the effect of gait training on balance outcomes. Considering body-weight-supported treadmill is not a common intervention in the chronic stroke stage (92, 93), treadmill might be an alternative choice.

In terms of static steady-state balance, our findings showed that no gait training was superior to the control intervention significantly. This finding is consistent with previous studies assessing the effect of gait training on stroke patients (40, 94). For example, a randomized controlled trial suggested that gait training with various weight shifts might have no significant effect on static postural control (95). In other words, balance capacity improved by dynamic tasks might have no effect on static balance. Meanwhile, it has been common knowledge that gait training mainly requires dynamic balance. Therefore, unsurprisingly, no type of gait training showed any effect on static steady-state balance.

Another interesting finding of our study was that gait training did not show a significant effect on proactive balance. Thus, this finding did not support our hypothesis that gait training was effective to improve proactive balance. A previous study reported that stroke patients’ capacity to recover effective mobility was only moderately associated with their capacity to improve proactive balance (96). This fact suggests that gait performance does not necessarily reflect post-stroke patients’ capacity to maintain proactive balance. However, the other studies reported significant improvement in the proactive balance of stroke patients after enhanced gait training (combining gait training with other task-oriented balance training) (66, 97). Therefore, the question of whether or not gait training has a positive effect on proactive balance is still open. The discrepancies between our results and some of those previously documented might be the consequence of the fact that most gait training included in our study is a single task rather than a combined task, resulting in a reduction in efficacy.

Our result indicated that virtual reality gait training was the optimal gait training for balance test batteries. One possible explanation is that VR provides a greater number of weight transfer opportunities in different directions than other types of gait training. Another explanation is that virtual reality training could mimic real barriers in the screen image and, thus, enabled the reproduction of a rhythmic weight shift on the paretic side (98). Our results concerning balance test batteries are similar to published research suggesting that virtual reality gait training could significantly improve the berg balance scale score (99). Moreover, a body-weight-supported treadmill was the second best gait training to improve balance test batteries of stroke patients. An explanation is that the body-weight-supported treadmill provides partial weight support, with which related training can be conducted at higher intensities (100). Meanwhile, high-intensity training is particularly effective in the rehabilitation of neurological diseases, including stroke (101).

There are several limitations to our study. First, missing evidence for some types of included gait training was identified in our NMA. For example, treadmill accounted for 26.8% of the intervention data. However, conventional gait training only accounted for 10.1%. Therefore, readers should take these results with caution due to inadequate data for direct comparisons for some types of gait training. Next, we failed to assess reactive balance in this NMA since few included trials reported reactive balance. Reactive balance is an important balance outcome to assess patients’ capacity to stabilize one’s body during perturbation. Future trials are supposed to consider reactive balance as an essential balance outcome when designing studies.

## Conclusion

In summary, this NMA suggested that gait training was effective to improve some types of balance outcomes of stroke patients. Considering body- weight-supported treadmill is not a common intervention in the chronic stroke stage (92, 93), treadmill is recommended for those who want to improve their dynamic steady-state balance. Virtual reality gait training is recommended for those who want to improve their balance test batteries. This study emphasizes the importance of gait training to improve the balance outcomes of stroke patients and guides clinicians to formulate better gait training prescriptions.

## Data availability statement

The original contributions presented in the study are included in the article/Supplementary material, further inquiries can be directed to the corresponding author.

## Author contributions

TL and YW: conceptualization. TL, KY, and JL: methodology. TL and XZ: formal analysis. TL and RW: investigation. XZ, RW, and CZ: resources. XZ, RW, CZ, and ML: data curation. TL and KY: writing—original draft preparation and editing. CL and CX: writing—reviewing. CZ and ML: visualization. YW: supervision. All authors contributed to the article and approved the submitted version.

## Funding

This study was supported by the National Key Research and Development Plan of China (2019YFC1710303).

## Conflict of interest

CX is employed by SUSE Software (Beijing) Co., Ltd.

The remaining authors declare that the research was conducted in the absence of any commercial or financial relationships that could be construed as a potential conflict of interest.

## Publisher’s note

All claims expressed in this article are solely those of the authors and do not necessarily represent those of their affiliated organizations, or those of the publisher, the editors and the reviewers. Any product that may be evaluated in this article, or claim that may be made by its manufacturer, is not guaranteed or endorsed by the publisher.

## Supplementary material

The Supplementary material for this article can be found online at: https://www.frontiersin.org/articles/10.3389/fneur.2023.1093779/full#supplementary-material
