# Supplementary figures and images for: Comparative efficacy of gait training for balance outcomes in patients with stroke: A systematic review and network meta-analysis
Source: Front Neurol. 2023 Apr 3;14:1093779. doi: 10.3389/fneur.2023.1093779 (PMC10106590; doi:10.3389/fneur.2023.1093779)

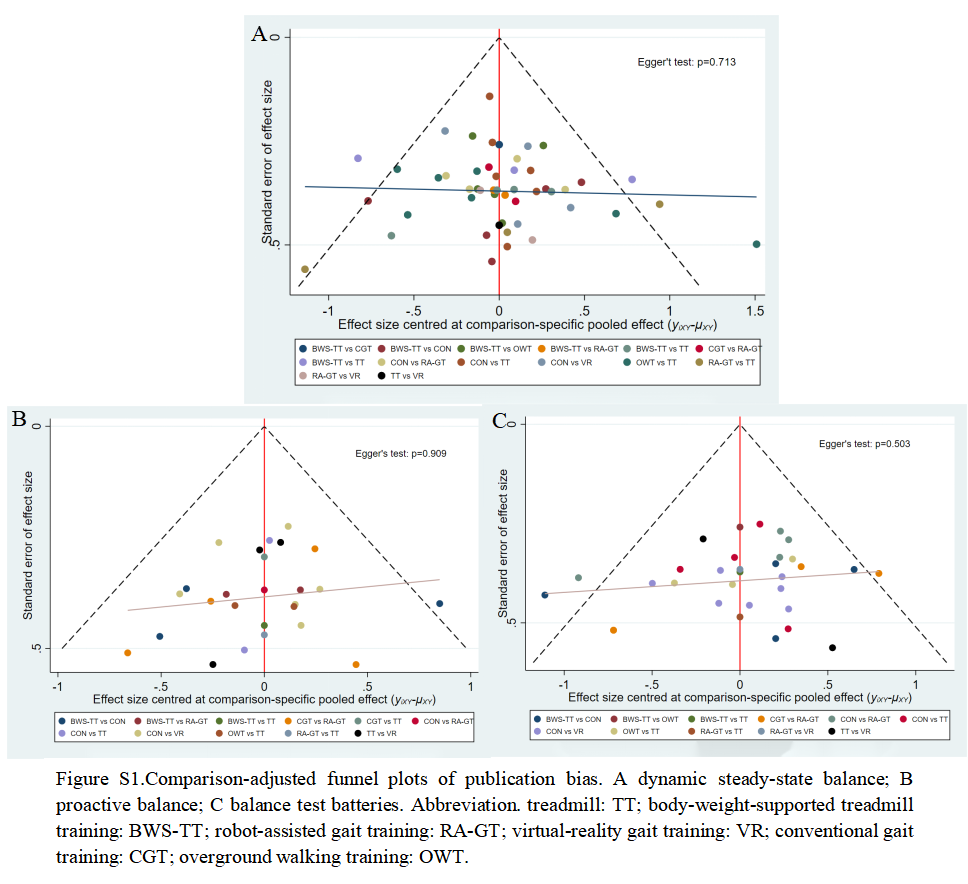

Supplement: Supplementary file 1 [file Image_1.TIF]

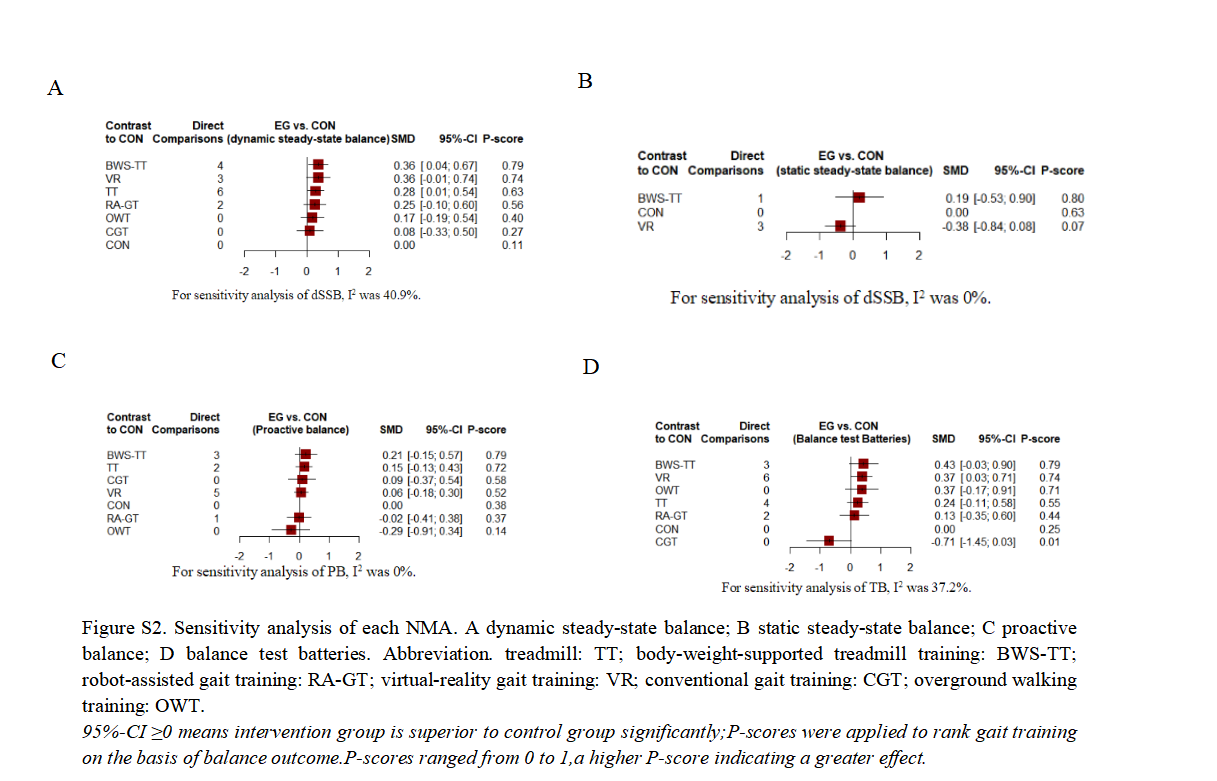

Supplement: Supplementary file 2 [file Image_2.TIF]
