# Supplementary material for: Comparative efficacy of gait training for balance outcomes in patients with stroke: A systematic review and network meta-analysis
Source: Front Neurol. 2023 Apr 3;14:1093779. doi: 10.3389/fneur.2023.1093779 (PMC10106590; doi:10.3389/fneur.2023.1093779)
Supplement: Supplementary file 3 [file Table_1.DOCX]

| #1 | Search: (((((((((((((((((((((((((((("Stroke"[Mesh]) OR (Strokes[Title/Abstract])) OR (Cerebrovascular Accident[Title/Abstract])) OR (Cerebrovascular Accidents[Title/Abstract])) OR (CVA[Title/Abstract])) OR (CVAs[Title/Abstract])) OR (Cerebrovascular Apoplexy[Title/Abstract])) OR (Apoplexy, Cerebrovascular[Title/Abstract])) OR (Vascular Accident, Brain[Title/Abstract])) OR (Brain Vascular Accident[Title/Abstract])) OR (Brain Vascular Accidents[Title/Abstract])) OR (Vascular Accidents, Brain[Title/Abstract])) OR (Cerebrovascular Stroke[Title/Abstract])) OR (Cerebrovascular Strokes[Title/Abstract])) OR (Stroke, Cerebrovascular[Title/Abstract])) OR (Strokes, Cerebrovascular[Title/Abstract])) OR (Apoplexy[Title/Abstract])) OR (Cerebral Stroke[Title/Abstract])) OR (Cerebral Strokes[Title/Abstract])) OR (Stroke, Cerebral[Title/Abstract])) OR (Strokes, Cerebral[Title/Abstract])) OR (Stroke, Acute[Title/Abstract])) OR (Acute Stroke[Title/Abstract])) OR (Acute Strokes[Title/Abstract])) OR (Strokes, Acute[Title/Abstract])) OR (Cerebrovascular Accident, Acute[Title/Abstract])) OR (Acute Cerebrovascular Accident[Title/Abstract])) OR (Acute Cerebrovascular Accidents[Title/Abstract])) OR (Cerebrovascular Accidents, Acute[Title/Abstract]) |
| --- | --- |
| #2 | Search: (((((((((((("Virtual Reality"[Mesh]) OR (Reality, Virtual[Title/Abstract])) OR (Virtual Reality, Educational[Title/Abstract])) OR (Educational Virtual Realities[Title/Abstract])) OR (Educational Virtual Reality[Title/Abstract])) OR (Reality, Educational Virtual[Title/Abstract])) OR (Virtual Realities, Educational[Title/Abstract])) OR (Virtual Reality, Instructional[Title/Abstract])) OR (Instructional Virtual Realities[Title/Abstract])) OR (Instructional Virtual Reality[Title/Abstract])) OR (Realities, Instructional Virtual[Title/Abstract])) OR (Reality, Instructional Virtual[Title/Abstract])) OR (Virtual Realities, Instructional[Title/Abstract]) |
| #3 | Search: (Treadmill*[Title/Abstract]) OR (tread mill*[Title/Abstract]) |
| #4 | Search: (((Robot assist*[Title/Abstract]) OR (robotic assist*[Title/Abstract])) OR (robotic gait[Title/Abstract])) OR (robotic intervention[Title/Abstract]) |
| #5 | Search: (((support*[Title/Abstract]) OR (suspen*[Title/Abstract])) OR (relief[Title/Abstract])) AND (((weight[Title/Abstract]) OR (body‐weight[Title/Abstract])) OR (bodyweight[Title/Abstract])) |
| #6 | Search: (conventional gait*[Title/Abstract]) OR (CGT*[Title/Abstract]) |
| #7 | Search: (overground walking*[Title/Abstract]) OR (OWT*[Title/Abstract]) |
| #8 | Search: #2 OR #3 OR #4 OR #5 OR #6 OR #7 |
| #9 | Search: (randomized controlled trial[pt] OR controlled clinical trial[pt] OR randomized[tiab] OR placebo[tiab] OR drug therapy[sh] OR randomly[tiab] OR trial[tiab] OR groups[tiab]) NOT (animals[mh] NOT humans[mh]) |
| #10 | Search: #1 AND #9 AND #10 |

**Table. S1 Search strategy for Pubmed**

| Author/Year  **Table. S2 Characteristic of included studies** | Country | Gender  (male) | Age(years,  mean±SD) | Disease duration (months,  mean ±SD) | Intervention  group | Control  group | Duration (weeks) | Frequency (x/week) | Time min/ repetitions | Outcome |
| --- | --- | --- | --- | --- | --- | --- | --- | --- | --- | --- |
| Nave et al. 2019^[29]^ | Germany | TT:105(60)  CON:95(59) | TT:(69±12) CON:(70±11) | TT:30(17-39)^*^  CON:27(17-41)^*^ | TT | CON | 4 | 5 | 25 | dSSB:MWS |
| Pohl et al. 2001^[30]^ | Germany | TT1:20(14) TT2:20(16)  CGT:20(13) | TT1(57.1±13.9) TT2(58.2±10.3)  CGT(61.6±10.6) | TT1(16.8±20.5)^#^ TT2(16.2±16.4)^#^  CGT(16.1±18.5^#^ | TT/CGT | NA | 4 | 6 | 30-45 | dSSB:MWS |
| Lee et al. 2019^[31]^ | Korea | TT:14(7) RA-GT:12(7) | TT(61.85±7.87) RA-GT(62.25±6.36) | TT(1486±264.12)  RA-GT(1536±311.54)* | TT/RA-GT | NA | 4 | 3 | 45 | dSSB:MWS |
| Graham et al. 2018^[32]^ | England | BWS-TT:15(8) TT:15(7) | TT-BWS(48.9±14.4) TT(60.3±12.8) | BWS-TT(52±71.4) TT(47.7±64.7) | BWS-TT/TT | NA | 6 | 3 | 30 | dSSB:MWS  TB:BBS |
| Cannell et al. 2017^[33]^ | Australia | VR:39(23) CON:40(25) | VR(72.8±10.4)  CON(74.8±11.9) | VR(19±13)* CON(26±27)* | VR | CON | 8 | 5 | 60 | dSSB:MWS  PB:TUG |
| Gjellesvik et al. 2021^[34]^ | Norway | TT:36(31) CON:34(20) | TT(57.6±9.2) CON(58.7±9.2) | TT(25.4±14.5) CON(27.4±14.7) | TT | CON | 8 | 4 | 25 | dSSB:10mWT  PB:TUG  TB:BBS |
| Globas et al. 2012^[35]^ | Switzerland | TT:18(14) CON:18(15) | TT(68.6±6.7) CON(68.7±6.1) | TT(60.2±46.6) CON(70±67.4) | TT | CON | 12 | 1-3 | 30-50 | dSSB:10mWT  TB:BBS |
| DePaul et al. 2015^[36]^ | Canada | BWS-TT:36(22)  OWT:35(21) | BWS-TT(69.03±12.26) OWT(66.40±10.98) | BWS-TT(18.5±19.81)^#^ OWT(18±14.81)^#^ | BWS-TT/OWT | NA | 5 | 3 | 30 | dSSB:MWS |
| Langhammer et al. 2015^[37]^ | Norway | TT:21(10)  OWT:18(6) | TT(74±13.3)  OWT(75±10.4) | TT(419±1034) OWT(349±820)* | TT/OWT | NA | not mentioned | 5 | 30 | dSSB:10mWT |
| Høyer et al. 2012^[38]^ | Norway | BWS-TT:30(20)  CGT:30(18) | BWS-TT(52.3±10.4)  CGT(52.0±13.1) | BWS-TT(99±39.4)  CGT(96±42.0)* | BWS-TT/CGT | NA | 10 | 1-7 | 30 | dSSB:10mWT |
| In et al. 2016^[39]^ | Korea | VR:13(8)  CON:13(7) | VR(57.31±10.53)  CON(54.42±11.44) | VR(12.54±4.14)  CON(13.58±5.28) | VR | CON | 4 | 3 | 30 | dSSB:10mWT  sSSB:EO-TS  PB:FRT  TB:BBS |
| Lloréns et al. 2015^[40]^ | Spain | VR:10(5)  CON:10(5) | VR(58.3±11.6)  CON(55.0±11.6) | VR(407.5±232.4)  CON(587.6±222.1)* | VR | CON | 4 | 5 | 60 | TB:BBS |
| Park et al. 2017^[41]^ | Korea | VR:8(5)  CON:8(6) | VR(52.39±6.06)  CON(54.38±14.05) | VR(10.78±7.06)  CON(14.10±7.73) | VR | CON | 6 | 7 | 30 | dSSB:10mWT  PB:TUG  TB:BBS |
| McEwen et al. 2014^[42]^ | Canada | VR:30(16)  CON:30(16) | VR(62.2±14.1)  CON(66.0±15.8) | VR(30.1±18.9)  CON(39.6±17.8)* | VR | CON | 4 | 3 | 30 | dSSB:2mWT  PB:TUG |
| Mirelman et al. 2009^[43]^ | USA | VR:9(7)  RA-GT:9(8) | VR(61.8±9.94)  RA-GT(61±8.32) | VR(37.7±25)  RA-GT(58.2±26.3) | VR/RA-GT | NA | 4 | 3 | 60 | dSSB:MWS |
| Lloréns et al. 2014^[44]^ | Spain | VR:15(10)  CON:15(7) | VR(55.47±9.63)  CON(55.60±7.29) | VR(334.13±60.79)  CON(316.73±49.81)* | VR | CON | 7 | 3 | 45 | TB:BBS |
| Rooij et al. 2021^[45]^ | Netherlands | VR:28(18)  TT:24(18) | VR(65±9.63)  TT(61±13.33) | VR(84±30.37)  TT(66±38.52)* | VR/TT | NA | 6 | 2 | 30 | PB:TUG  TB:miniBEST |
| Kayabinar et al. 2021^[46]^ | Turkey | VR:15(10)  RA-GT:15(8) | VR(58.80±5.03)  RA-GT(57.06±6.75) | VR(20±58.52)  RA-GT(36±48.15) | VR/RA-GT | NA | 6 | 2 | 45 | dSSB:10mWT  TB:BBS |
| Lee et al. 2018^[47]^ | Korea | VR:15(9)  CON:15(9) | VR(61.80±6.80)  CON(61.33±8.44) | VR(3.43±1.34)  CON(3.13±1.54) | VR | CON | 5 | 3 | 30 | sSSB:EO-CoP PL  PB:FRT |
| Tramontano et al. 2018^[48]^ | Italy | VR:13(8)  CON:12(8) | VR(63.1±8.5)  CON(65.1±15.5) | not mentioned | VR | CON | 4 | 3 | 20 | PB:FRT |
| Kim et al. 2020^[49]^ | Korea | RA-GT:14(11)  BWS-TT:14(12) | RA-GT(55±12)  BWS-TT(51±10) | RA-GT(6.14±2.83)  BWS-TT(5.79±2.67) | RA-GT/BWS-TT | NA | 4 | 5 | 30 | dSSB:10mWT  PB:TUG |
| Yu et al. 2021^[50]^ | China | RA-GT:27(18)  CGT:27(12) | RA-GT(57.89±10.08)  CGT(52.11±5.49) | RA-GT(7.00±2.12)  CGT(7.89±2.57)^#^ | RA-GT/CGT | NA | 2 | 7 | 120 | PB:TUG |
| Santos et al. 2018^[51]^ | Brazil | RA-GT:7(5)  CGT:8(6) | RA-GT(44.4±12.7)  CGT(56.4±11.8) | RA-GT(4.8±0.92)  CGT(10.5±5.4)^$^ | RA-GT/CGT | NA | 3 | 3 | 60 | PB:TUG  TB:BBS |
| YUN et al. 2018^[52]^ | Korea | RA-GT:18(10)  CON:18(9) | RA-GT(63.6±8.3)  CON(64.3±8.4) | RA-GT(31.3±7.5)  CON(28.8±6.8)* | RA-GT | CON | 3 | 5 | 30 | TB:BBS |
| Irene et al. 2019^[53]^ | Italy | RA-GT:14(10)  CGT:12(9) | RA-GT(56.43±12.93)  CGT(61.58±9) | RA-GT(44.92±16.02)  CGT(86.58±52.84) | RA-GT/CGT | NA | 7 | 3 | 45 | dSSB:CWS |
| Bang et al. 2016^[54]^ | Korea | RA-GT:9(5)  TT:9(4) | RA-GT(53.56±3.94)  TT(53.67±2.83) | RA-GT(11.56±2.60)  TT(12.56±2.65) | RA-GT/TT | NA | 4 | 5 | 60 | dSSB:CWS  TB:BBS |
| Li et al. 2021^[55]^ | China | RA-GT:17(15)  CON:15(14) | RA-GT(50.53±12.26)  CON(50.13±9.49) | RA-GT(2.53±1.33)  CON(3.38±1.19) | RA-GT | CON | 4 | 2 | 30 | dSSB:10mWT |
| Naojiro et al. 2018^[56]^ | Japan | RA-GT:21(13)  CGT:20(14) | RA-GT(64.9±12.2)  CGT(62.3±9.3) | RA-GT(103.9±28.1)^*^  CGT(92.9±35.9^*^ | RA-GT/CGT | NA | not mentioned | not mentioned | 30 | dSSB:MWS |
| Ogino et al. 2020^[57]^ | Japan | RA-GT:8(6)  TT:11(9) | RA-GT(66.1±9.6)  TT(65.0±7.7) | RA-GT(8.0±5.1)  TT(7.1±4.1)^$^ | RA-GT/TT | NA | 4 | 5 | 40 | dSSB:CWS  PB:TUG |
| Han et al. 2016^[58]^ | Korea | RA-GT:30(17)  CON:26(15) | RA-GT(67.89±14.96)  CON(63.2±10.62) | RA-GT(21.56±7.98)  CON(18.10±9.78)* | RA-GT | CON | 4 | 5 | 60 | TB:BBS |
| Srivastava et al. 2016^[59]^ | India | BWS-TT:15(12)  TT:15(12) | BWS-TT(44.20±11.70)  TT(47.93±9.95) | BWS-TT(391.80±431.10)  TT(442.07±295.13)* | BWS-TT/TT | NA | 4 | 5 | 30 | dSSB:CWS |
| Gama et al. 2016^[60]^ | Brazil | BWS-TT:14(7)  OWT:14(8) | BWS-TT(58.7±8.4)  OWT(57.7±10.1) | BWS-TT(60.2±55.4)  OWT(53.8±42.2) | BWS-TT/OWT | NA | 6 | 3 | not mentioned | dSSB:10mWT |
| Yen et al. 2007^[61]^ | Korea | BWS-TT:7(3)  OWT:7(6) | BWS-TT(57.30±16.44)  OWT(56.05±12.69) | BWS-TT(1.97±0.61)  OWT(1.96±2.42)^$^ | BWS-TT | CON | 4 | 3 | 30 | dSSB:CWS  TB:BBS |
| Kang et al. 2011^[62]^ | Korea | BWS-TT:10(6)  TT:10(4) | BWS-TT(55.9±6.5)  TT(56.3±7.6) | BWS-TT(14.1±4.4)  TT(13.5±4.0) | BWS-TT/TT | NA | 4 | 3 | 30 | dSSB:10mWT  PB:FRT |
| Kuys et al. 2015^[63]^ | Australia | TT:14(7)  CON:15(5) | TT(63±14)  CON(72±17) | TT(52±32)*  CON(49±30)* | TT | CON | 6 | 3 | 30 | dSSB:MWS |
| Palmcrantz .et al 2021^[64]^ | Sweden | BWS-TT:16(11)  CON:17(11) | BWS-TT(62.25±7.9)  CON(61.65±8.59) | BWS-TT(21±18.33)  CON(38±25.56) | BWS-TT | CON | 6 | 3 | 30 | dSSB:10mWT  TB:BBS |
| Aguiar et al. 2020^[65]^ | Canada | TT:11(8)  OWT:11(8) | TT(52±11)  OWT(48±10) | TT(51±68)  OWT(44±26) | TT/OWT | NA | 12 | not mentioned | 40 | dSSB:MWS |
| Baer et al. 2017^[72]^ | UK | TT:38(18)  CGT:39(22) | TT(74.5±11.7)  CGT(71.23±12.52) | TT(40.26±21.43)  CGT(42.13±19.48)* | TT/CGT | NA | 8 | 2 | not mentioned | dSSB:MWS  PB:TUG |
| Manji et al. 2018^[66]^ | Japan | BWS-TT:15(10)  RA-GT:15(11) | BWS-TT(62.2±10.1)  RA-GT(63.7±11) | BWS-TT(134.5±55.7)  RA-GT(149.7±24.2)* | BWS-TT/RA-GT | NA | 1 | 7 | 20 | dSSB:MWS  PB:TUG |
| Lamberti.et al. 2017^[67]^ | Italy | TT:17(13)  OWT:18(14) | TT(67±10)  OWT(69±9) | TT(40±51)  OWT(34±46) | TT/OWT | NA | 8 | 3 | 60 | dSSB:10mWT  TB:BBS |
| Kim et al. 2017^[68]^ | Korea | BWS-TT:15(11)  TT:15(9) | BWS-TT(48.27±16.05)  TT(50.73±13.50) | BWS-TT(10.93±3.67)  TT(11.27±4.10) | BWS-TT/TT | NA | 4 | 5 | 30 | dSSB:CWS |
| Danks et al. 2016^[69]^ | USA | TT:13(7)  OWT:14(8) | TT(59.1±8.7)  OWT(58.2±12.4) | TT(29.4±21.4)  OWT(50.8±44.1) | TT/OWT | NA | 12 | 3 | 30 | dSSB:MWS |
| Hollands et al. 2015^[70]^ | UK | TT:18(11)  OWT:19(14)  CON:19(8) | TT(59±18)  OWT(56.1±12.2)  CON(60±13.6) | TT(7.8±15.4)  OWT(8.6±11.3)  CON(7.8±11.1) | TT/OWT | CON | 8 | 2 | 30 | dSSB:CWS  PB:TUG  TB:BBS |
| Miller. et al. 2014^[71]^ | USA | BWS-TT:10(4)  OWT:10(7) | BWS-TT(56.20±7.61)  OWT(65.50±6.17) | BWS-TT(62.30±48.64)  OWT(60.00±51.68) | BWS-TT/OWT | NA | 2 | 5 | 30 | dSSB:MWS |
| Dobkin et al. 2014^[78]^ | USA | TT:15  CON:15 | Not mentioned | Not mentioned | TT | CON | 12 | 3 | 90 | PB:TUG |
| Cho et al.2013^[73]^ | Korea | VR:7(3)  TT:7(4) | VR(64.57±4.35)  TT(65.14±4.74) | VR(288.28±69.20)  TT(312.42±83.68)* | VR/TT | NA | 6 | 3 | 30 | PB:TUG  TB:BBS |
| Olawale et al. 2011^[74]^ | USA | TT:20(12)  OWT:20(11)  CON:20(11) | TT(56.8±6.4)  OWT(56.8±8.3)  CON(57.2±5.9) | TT(10.2±6.9)  OWT(10.7±6.8)  CON(10.3±5.9) | TT/OWT | CON | 12 | 3 | 60 | dSSB:CWS |
| Yang et al. 2008^[75]^ | China | VR:9(5)  TT:11(5) | VR(60.89±9.25)  TT(55.45±12.15) | VR(6.10±10.32)  TT(5.93±4.17) | VR/TT | NA | 3 | 3 | 20 | dSSB:CWS |
| Nilsson et al. 2001^[76]^ | Sweden | BWS-TT:36(20)  OWT:37(20) | BWS-TT(54±31.85)  OWT(56±31.11) | BWS-TT(22±34.07)  OWT(17±33.33)* | BWS-TT/OWT | NA | 4 | 5 | 30 | dSSB:CWS  TB:BBS |
| Miclaus et al. 2021^[77]^ | UK | VR:31(7)  TT:28(8) | VR(59.03±10.12)  TT(60.67±8.17) | VR(2.74±1.1)  TT(2.71±0.99) | VR/TT | NA | not mentioned | not mentioned | 70 | PB:FRT |
| Song et al. 2014^[79]^ | Korea | VR:10(4)  CON:10(5) | VR(65.6±13.5)  CON(60.6±18.2) | VR(12.7±3.2)  CON(12.8±3.4)* | VR | CON | 3 | 5 | 25 | dSSB:CWS  PB:TUG |
| Kang et al. 2021^[80]^ | Korea | RA-GT:15(10)  CON:15(8) | RA-GT(64.3±4.6)  CON(62.9±6.0) | not mentioned | RA-GT | CON | 3 | 3 | 30 | dSSB:CWS  PB:TUG  TB:BBS |
| K-Song. et al. 2021^[81]^ | Korea | RA-GT:18(12)  CON:18(9) | RA-GT(61.22±12.78)  CON(60.39±13.59) | RA-GT(3.4±3.6)  CON(3.6±5.4) | RA-GT | CON | 3 | 5 | 30 | dSSB:CWS  TB:BBS |
| Park et al. 2020^[82]^ | USA | RA-GT:7(4)  CGT:7(6) | RA-GT(69.86)  CGT(76.29) | not mentioned | RA-GT/CGT | NA | 2 | 7 | 30 | TB:BBS |
| B-Santos et al. 2018^[83]^ | Brazil | RA-GT:7(5)  CGT:8(6) | RA-GT(44.4±12.7)  CGT(56.4±11.8) | RA-GT(4.8±0.92)  CGT(10.5±5.4)^$^ | RA-GT/CGT | NA | not mentioned | not mentioned | not mentioned | PB:TUG  TB:BBS |
| Gangopadhyay et al. 2021^[89]^ | India | BWS-TT:15(8)  CON:15(9) | BWS-TT(52.07±3.67)  CON(52.40±3.91) | not mentioned | BWS-TT | CON | 4 | 3 | 20 | dSSB:CWS  PB:TUG  TB:BBS |
| Kaiser et al. 2019^[84]^ | Germany | BWS-TT:9(6)  CON:9(7) | BWS-TT(63)  CON(66) | BWS-TT(62)  CON(102) | BWS-TT | CON | 6 | 5 | 30 | dSSB:CWS  PB:TUG |
| Mustafaoğlu et al. 2018^[85]^ | Turkey | BWS-TT:15(10)  CON:15(11) | BWS-TT(52.8±13.8)  CON(52.6±14.7) | BWS-TT(12.5±24.44)  CON(11±17.78) | BWS-TT | CON | 6 | 2 | 45 | dSSB:10mWT  PB:TUG  TB:BBS |
| Peurala.et al. 2005^[86]^ | Finland | BWS-TT:20(11)  OWT:20(11) | BWS-TT(51.2±7.9)  OWT(52.3±6.8) | BWS-TT(2.4±2.6)  OWT(4±5.8)^$^ | BWS-TT | CON | 3 | 5 | 20 | dSSB:10mWT  sSSB:COP |
| Marques-Sule et al.2021^[87]^ | Spain | TC:15(9)  CON:14(9) | TC(61.5±8.4)  CON(58.2±7.4) | not mentioned | VR | CON | 4 | 2 | 30 | PB:TUG  TB:BBS |
| Kim et al. 2018^[88]^ | Korea | RA-GT:25(20)  CON:23(13) | RA-GT(57.7±12.9)  CG(60.4±13.2) | RA-GT(2.0±2.4)  CON(2.6±3.1) | RA-GT | CON | 3 | 5 | 30 | dSSB:10mWT  TB:BBS |

Note: *days,#weeks,$years. MWS max walking speed. CWS comfortable walking speed. 10mWT 10 meter waking test. 2mWT 2 meter walking speed. DGI dynamic gait index. SB sitting balance. EO eyes open. TS total sway distance. SL sway length. TEC time on one foot standing with eyes closed. CoP center of pressure;PL-path length. FRT functional reach test. TUG time up-and-go test. BBS berg balance scale.treadmill: TT; body-weight-supported treadmill training: BWS-TT; robot-assisted gait training: RA-GT; virtual-reality gait training: VR; conventional gait training: CGT; overground walking training: OWT; CON: control group.

| **Fig. S1 Risk of bias graph for each included study**  **ID** | **Author/Year** | **Randomization process** | **Deviations from intended interventions** | **Missing outcome data** | **Measurement of the outcome** | **Selection of the reported result** |
| --- | --- | --- | --- | --- | --- | --- |
| 1 | Nave et al. | Low | Low | Low | Low | Low |
| 2 | Pohl et al. 2001 | Low | Low | Low | Low | Low |
| 3 | Lee et al.2018 | Low | Some concerns | Low | Low | Low |
| 4 | Graham et al.2018 | Low | Low | Low | Low | Low |
| 5 | Cannell et al.2017 | Low | Low | Low | Low | Low |
| 6 | Gjellesvik et al.2021 | Low | Some concerns | Low | Low | Low |
| 7 | Globas et al.2012 | Low | Low | Low | Low | Low |
| 8 | DePaul et al.2015 | Low | Low | Some concerns | Low | Low |
| 9 | Langhammer et al.2015 | Low | Some concerns | Some concerns | Low | Some concerns |
| 10 | Høyer et al.2012 | Low | Some concerns | Low | Low | Low |
| 11 | In et al.2016 | Low | Some concerns | Low | Low | Some concerns |
| 12 | Llorens et al.2015 | Low | Some concerns | Some concerns | Low | Low |
| 13 | Park et al.2017 | Low | High | High | Low | Low |
| 14 | Zheng et al.2019 | Low | Low | Low | Low | Low |
| 15 | McEwen et al.2014 | Low | Some concerns | Some concerns | Low | Low |
| 16 | Mirelman et al.2009 | Some concerns | Some concerns | Low | Low | Some concerns |
| 17 | Lloréns et al.2014 | Low | Low | Low | Low | Low |
| 18 | Rooij et al. 2021 | Low | Low | Low | Low | Low |
| 19 | Kayabinar et al.2021 | Low | Some concerns | Low | Low | Low |
| 20 | Lee et al | Low | Some concerns | Low | Low | Low |
| 21 | Tramontano et al.2018 | Some concerns | Some concerns | Low | Low | Low |
| 22 | Kim et al. | Low | Some concerns | Low | Low | Low |
| 23 | Deng et al.2021 | Low | Some concerns | Low | Low | Low |
| 24 | Santos et al.2018 | High | Some concerns | Low | Low | Low |
| 25 | Yun et al. | Low | Some concerns | Low | Low | Low |
| 26 | Irene et al.2019 | Low | Low | Low | Low | Low |
| 27 | Bang et al.2016 | Low | Some concerns | Low | Low | Low |
| 28 | Li et al.2021 | Low | Some concerns | Low | Low | Low |
| 29 | Naojiro et al.2018 | Some concerns | Low | Low | Low | Low |
| 30 | Ogino et al.2020 | Low | Some concerns | Low | Low | Low |
| 31 | Han et al.2016 | Low | Some concerns | Low | Low | Low |
| 32 | Srivastava et al.2016 | Low | Some concerns | Low | Low | Low |
| 33 | Gama et al.2016 | Low | Low | Low | Low | Low |
| 34 | Yen et al.2007 | Low | Low | Low | Low | Low |
| 35 | Kang et al.2011 | Low | Some concerns | Low | Low | Low |
| 36 | Kuys et al.2015 | Low | Low | Low | Low | Low |
| 37 | Palmcrantz et al.2021 | Low | High | High | Low | Low |
| 38 | Aguiar et al.2020 | Low | Low | Low | Low | Low |
| 39 | Baer et al.2017 | Low | Low | Low | Low | Low |
| 40 | Manji et al.2018 | Low | Low | Low | Low | Low |
| 41 | Lamberti et al.2017 | Low | Some concerns | Low | Low | Low |
| 42 | Kim et al.2017 | Low | Some concerns | Low | Low | Low |
| 43 | Danks et al.2016 | Low | Some concerns | Low | Low | Low |
| 44 | Hollands et al.2015 | Low | Some concerns | Low | Low | Low |
| 45 | Miller et al. | Some concerns | Some concerns | Low | Low | Low |
| 46 | Dobkin et al.2014 | Some concerns | Low | Low | Low | Low |
| 47 | Cho et al.2013 | Some concerns | Some concerns | Low | Low | Low |
| 48 | Olawale et al. | Low | Some concerns | Low | Low | Low |
| 49 | Yang et al.2008 | Low | Some concerns | Low | Low | Low |
| 50 | Nilsson et al.2001 | Low | High | Low | Low | Low |
| 51 | Miclaus et al.2021 | Low | Some concerns | Low | Low | Low |
| 52 | Song et al.2014 | Some concerns | Some concerns | Low | Low | Low |
| 53 | Kang et al.2021 | Low | Low | Low | Low | Low |
| 54 | Song et al.2021 | Low | Some concerns | Some concerns | Low | Low |
| 55 | Park et al. | Some concerns | Some concerns | Low | Low | Low |
| 56 | Santos et al.2018 | High | High | High | Low | Low |
| 57 | Gangopadhyay et al.2021 | Low | Low | Low | Low | Low |
| 58 | Kaiser et al.2019 | Low | Some concerns | Low | Low | Low |
| 59 | Mustafaoğlu et al.2018 | Low | Low | Low | Low | Low |
| 60 | Peurala et al.2005 | Low | Some concerns | Low | Low | Low |
| 61 | kim et al.2019 | Some concerns | Some concerns | Some concerns | Low | Low |

| Design-specific decomposition of within-designs Q statistic  **Table. S3A Design-based decomposition of Cochran’s Q of dynamic steady-state balance** | | | | | |
| --- | --- | --- | --- | --- | --- |
| Design | Q | df | p-value |  |  |
| BWS-TT vs OWT | 1.55 | 4 | 0.8184 |  |  |
| BWS-TT vs RA-GT | 0.02 | 1 | 0.8993 |  |  |
| BWS-TT vs TT | 2.49 | 3 | 0.4777 |  |  |
| CGT vs RA-GT | 0.1 | 1 | 0.7562 |  |  |
| CGT vs TT | 13.17 | 2 | 0.0014 |  |  |
| CON vs BWS-TT | 6.3 | 4 | 0.178 |  |  |
| CON vs RA-GT | 2.34 | 3 | 0.5044 |  |  |
| CON vs TT | 0.87 | 5 | 0.9724 |  |  |
| CON vs VR | 3.48 | 3 | 0.3232 |  |  |
| OWT vs TT | 17.95 | 6 | 0.0064 |  |  |
| RA-GT vs TT | 9.23 | 2 | 0.0099 |  |  |
| RA-GT vs VR | 0.25 | 1 | 0.6173 |  |  |
| Between-designs Q statistic after detaching of single designs | | | | | |
| Detached design | Q | df | p-value |  |  |
| BWS-TT vs CGT | 10.72 | 7 | 0.1511 |  |  |
| BWS-TT vs OWT | 6.49 | 7 | 0.484 |  |  |
| BWS-TT vs RA-GT | 10.1 | 7 | 0.1829 |  |  |
| BWS-TT vs TT | 7.97 | 7 | 0.3357 |  |  |
| CGT vs RA-GT | 11.07 | 7 | 0.1354 |  |  |
| CGT vs TT | 10.8 | 7 | 0.1478 |  |  |
| CON vs BWS-TT | 11.07 | 7 | 0.1355 |  |  |
| CON vs RA-GT | 10.21 | 7 | 0.1768 |  |  |
| CON vs TT | 10.98 | 7 | 0.1396 |  |  |
| CON vs VR | 7.9 | 7 | 0.3413 |  |  |
| OWT vs TT | 6.49 | 7 | 0.484 |  |  |
| RA-GT vs TT | 9.76 | 7 | 0.2028 |  |  |
| RA-GT vs VR | 8.56 | 7 | 0.2859 |  |  |
| TT vs VR | 10.69 | 7 | 0.1529 |  |  |
| Q statistic to assess consistency under the assumption of a full design-by-treatment interaction random effects model | | | | | |
|  | Q | df | p-value | tau.within | tau2.within |
| Between designs | 7.71 | 8 | 0.4625 | 0.2778 | 0.0772 |

Note: treadmill: TT; body-weight-supported treadmill training: BWS-TT; robot-assisted gait training: RA-GT; virtual-reality gait training: VR; conventional gait training: CGT; overground walking training: OWT; CON: control group.

**Table. S3B Design-based decomposition of Cochran’s Q of static steady-state balance**

| Design-specific decomposition of within-designs Q statistic | | | | | |
| --- | --- | --- | --- | --- | --- |
| Design | Q | df | p-value |  |  |
| CON vs VR | 0.61 | 2 | 0.7353 |  |  |
| Between-designs Q statistic after detaching of single designs | | | | | |
|  | | | | | |
| Q statistic to assess consistency under the assumption of a full design-by-treatment interaction random effects model | | | | | |
|  | Q | df | p-value | tau.within | tau2.within |
| Between designs | 0.00 | 0 |  | 0 | 0 |

Note: virtual-reality gait training: VR; CON: control group.

**Table. S3C Design-based decomposition of Cochran’s Q of proactive balance**

| Design-specific decomposition of within-designs Q statistic | | | | | |
| --- | --- | --- | --- | --- | --- |
| Design | Q | df | p-value |  |  |
| BWS-TT vs RA-GT | 0.46 | 1 | 0.4961 |  |  |
| CGT vs RA-GT | 3.59 | 3 | 0.3097 |  |  |
| CON vs BWS-TT | 6.75 | 2 | 0.0342 |  |  |
| CON vs TC | 5.14 | 1 | 0.0234 |  |  |
| CON vs TT | 0.05 | 1 | 0.8298 |  |  |
| CON vs VR | 2.99 | 5 | 0.7014 |  |  |
| OWT vs TT | 0.25 | 1 | 0.6172 |  |  |
| TT vs VR | 0.31 | 2 | 0.8548 |  |  |
| Between-designs Q statistic after detaching of single designs | | | | | |
| Detached design | Q | df | p-value |  |  |
| BWS-TT vs RA-GT | 2.63 | 4 | 0.6217 |  |  |
| BWS-TT vs TT | 2.57 | 4 | 0.6319 |  |  |
| CGT vs RA-GT | 2.49 | 4 | 0.6457 |  |  |
| CGT vs TT | 2.49 | 4 | 0.6457 |  |  |
| CON vs BWS-TT | 2.14 | 4 | 0.7097 |  |  |
| CON vs RA-GT | 1.93 | 4 | 0.7494 |  |  |
| CON vs TT | 2.83 | 4 | 0.5875 |  |  |
| CON vs VR | 2.82 | 4 | 0.588 |  |  |
| RA-GT vs TT | 1.25 | 4 | 0.8692 |  |  |
| TT vs VR | 2.82 | 4 | 0.588 |  |  |
| Q statistic to assess consistency under the assumption of a full design-by-treatment interaction random effects model | | | | | |
|  | Q | df | p-value | tau.within | tau2.within |
| Between designs | 2.86 | 5 | 0.7220 | 0 | 0 |

Note: treadmill: TT; body-weight-supported treadmill training: BWS-TT; robot-assisted gait training: RA-GT; virtual-reality gait training: VR; conventional gait training: CGT; overground walking training: OWT; CON: control group.

**Table. S3D Design-based decomposition of Cochran’s Q of balance test batteries**

| Design-specific decomposition of within-designs Q statistic | | | | | |
| --- | --- | --- | --- | --- | --- |
| Design | Q | df | p-value |  |  |
| CGT vs RA-GT | 8.51 | 3 | 0.0365 |  |  |
| CON vs BWS-TT | 10.24 | 3 | 0.0166 |  |  |
| CON vs RA-GT | 7.62 | 3 | 0.0545 |  |  |
| CON vs TT | 1.36 | 3 | 0.7142 |  |  |
| CON vs VR | 2.79 | 6 | 0.8352 |  |  |
| OWT vs TT | 1.66 | 2 | 0.4363 |  |  |
| TT vs VR | 1.35 | 1 | 0.2446 |  |  |
| Between-designs Q statistic after detaching of single designs | | | | | |
| Detached design | Q | df | p-value |  |  |
| BWS-TT vs OWT | 3.59 | 4 | 0.4642 |  |  |
| BWS-TT vs TT | 3.17 | 4 | 0.5295 |  |  |
| CON vs BWS-TT | 3.11 | 4 | 0.5392 |  |  |
| CON vs RA-GT | 0.85 | 4 | 0.9317 |  |  |
| CON vs TT | 3.46 | 4 | 0.4833 |  |  |
| CON vs VR | 3.5 | 4 | 0.4776 |  |  |
| OWT vs TT | 3.59 | 4 | 0.4642 |  |  |
| RA-GT vs TT | 1.43 | 4 | 0.8386 |  |  |
| RA-GT vs VR | 2.97 | 4 | 0.563 |  |  |
| TT vs VR | 3.57 | 4 | 0.4678 |  |  |
| Q statistic to assess consistency under the assumption of a full design-by-treatment interaction random effects model | | | | | |
|  | Q | df | p-value | tau.within | tau2.within |
| Between designs | 2.71 | 5 | 0.7446 | 0.2989 | 0.0893 |

Note: treadmill: TT; body-weight-supported treadmill training: BWS-TT; robot-assisted gait training: RA-GT; virtual-reality gait training: VR; conventional gait training: CGT; overground walking training: OWT; CON: control group.

**Table. S4A Node-splitting analysis of dynamic steady-state balance**

| comparison | k | prop | nma | direct | indir. | Diff | z | p-value |
| --- | --- | --- | --- | --- | --- | --- | --- | --- |
| BWS-TT vs CGT | 1 | 0.28 | -0.25 | -0.09 | -0.31 | 0.22 | 0.5 | 0.6176 |
| BWS-TT vs CON | 5 | 0.43 | -0.3 | -0.3 | -0.29 | 0 | -0.01 | 0.9926 |
| BWS-TT vs OWT | 5 | 0.61 | -0.16 | 0.06 | -0.51 | 0.56 | 1.88 | 0.0603 |
| BWS-TT vs RA-GT | 2 | 0.27 | -0.08 | -0.3 | 0 | -0.3 | -0.8 | 0.4211 |
| BWS-TT vs TT | 4 | 0.31 | -0.05 | -0.34 | 0.09 | -0.43 | -1.5 | 0.1334 |
| BWS-TT vs VR | 0 | 0 | 0.04 | NA | 0.04 | NA | NA | NA |
| CGT vs CON | 0 | 0 | -0.05 | NA | -0.05 | NA | NA | NA |
| CGT vs OWT | 0 | 0 | 0.08 | NA | 0.08 | NA | NA | NA |
| CGT vs RA-GT | 2 | 0.41 | 0.17 | 0.14 | 0.19 | -0.05 | -0.11 | 0.9092 |
| CGT vs TT | 3 | 0.56 | 0.2 | 0.28 | 0.09 | 0.19 | 0.52 | 0.6065 |
| CGT vs VR | 0 | 0 | 0.29 | NA | 0.29 | NA | NA | NA |
| OWT vs CON | 0 | 0 | -0.13 | NA | -0.13 | NA | NA | NA |
| RA-GT vs CON | 4 | 0.47 | -0.22 | -0.31 | -0.14 | -0.17 | -0.59 | 0.5557 |
| TT vs CON | 6 | 0.59 | -0.25 | -0.29 | -0.19 | -0.1 | -0.4 | 0.6928 |
| VR vs CON | 4 | 0.7 | -0.34 | -0.18 | -0.7 | 0.52 | 1.36 | 0.1723 |
| OWT vs RA-GT | 0 | 0 | 0.09 | NA | 0.09 | NA | NA | NA |
| OWT vs TT | 7 | 0.66 | 0.12 | 0.31 | -0.25 | 0.56 | 1.88 | 0.0603 |
| OWT vs VR | 0 | 0 | 0.2 | NA | 0.2 | NA | NA | NA |
| RA-GT vs TT | 3 | 0.25 | 0.03 | -0.29 | 0.13 | -0.42 | -1.17 | 0.2425 |
| RA-GT vs VR | 2 | 0.33 | 0.12 | 0.47 | -0.06 | 0.52 | 1.22 | 0.2214 |
| TT vs VR | 1 | 0.14 | 0.09 | 0.29 | 0.05 | 0.24 | 0.42 | 0.6771 |

| comparison | k | prop | nma | direct | indir. | Diff | z | p-value |
| --- | --- | --- | --- | --- | --- | --- | --- | --- |
| BWS-TT vs CON | 1 | 1 | -0.1867 | -0.1867 | NA | NA | NA | NA |
| BWS-TT vs VR | 0 | 0 | -0.5661 | NA | -0.5661 | NA | NA | NA |
| VR vs CON | 3 | 1 | 0.3794 | 0.3794 | NA | NA | NA | NA |

Note: treadmill: TT; body-weight-supported treadmill training: BWS-TT; robot-assisted gait training: RA-GT; virtual-

reality gait training: VR; conventional gait training: CGT; overground walking training: OWT; CON: control group.

**Table. S4B Node-splitting analysis of static steady-state balance**

Note: body-weight-supported treadmill training: BWS-TT; CON: control group.

**Table. S4C Node-splitting analysis of proactive balance**

| comparison | k | prop | nma | direct | indir. | Diff | z | p-value |
| --- | --- | --- | --- | --- | --- | --- | --- | --- |
| BWS-TT vs CGT | 0 | 0 | -0.0868 | NA | -0.0868 | NA | NA | NA |
| BWS-TT vs CON | 3 | 0.62 | -0.2053 | -0.3275 | -0.0098 | -0.3177 | -0.82 | 0.4143 |
| BWS-TT vs OWT | 0 | 0 | -0.4927 | NA | -0.4952 | NA | NA | NA |
| BWS-TT vs RA-GT | 2 | 0.58 | -0.2424 | -0.1606 | -0.3483 | 0.1877 | 0.45 | 0.6549 |
| BWS-TT vs TT | 1 | 0.20 | -0.0554 | 0.1584 | -0.1100 | 0.2684 | 0.53 | 0.5929 |
| BWS-TT vs VR | 0 | 0 | -0.1585 | NA | -0.1585 | NA | NA | NA |
| CGT vs CON | 0 | 0 | -0.1207 | NA | -0.1207 | NA | NA | NA |
| CGT vs OWT | 0 | 0 | -0.4081 | NA | -0.4081 | NA | NA | NA |
| CGT vs RA-GT | 4 | 0.8 | -0.1578 | -0.2103 | 0.0466 | -0.2569 | -0.60 | 0.5469 |
| CGT vs TT | 1 | 0.53 | 0.0292 | 0.1512 | -0.1056 | 0.2569 | 0.60 | 0.5469 |
| CGT vs VR | 0 | 0 | -0.0739 | NA | -0.0739 | NA | NA | NA |
| OWT vs CON | 0 | 0 | 0.2874 | NA | 0.2874 | NA | NA | NA |
| RA-GT vs CON | 1 | 0.29 | 0.0371 | 0.3358 | -0.0868 | 0.4226 | 0.96 | 0.3346 |
| TT vs CON | 2 | 0.39 | -0.1499 | -0.1820 | -0.1296 | -0.0524 | -0.18 | 0.8578 |
| VR vs CON | 6 | 0.8 | -0.0468 | -0.0360 | -0.0900 | 0.0540 | 0.19 | 0.8518 |
| OWT vs RA-GT | 0 | 0 | 0.2503 | NA | 0.2503 | NA | NA | NA |
| OWT vs TT | 2 | 1 | 0.4373 | 0.4373 | NA | NA | NA | NA |
| OWT vs VR | 0 | 0 | 0.3342 | NA | 0.3342 | NA | NA | NA |
| RA-GT vs TT | 1 | 0.19 | 0.1813 | -0.3512 | 0.3024 | -0.6536 | -1.23 | 0.2174 |
| RA-GT vs VR | 0 | 0 | 0.0839 | NA | 0.0839 | NA | NA | NA |
| TT vs VR | 3 | 0.62 | -0.1032 | -0.0824 | -0.1364 | 0.0540 | 0.19 | 0.8518 |

Note: treadmill: TT; body-weight-supported treadmill training: BWS-TT; robot-assisted gait training: RA-GT; virtual-reality gait training: VR; conventional gait training: CGT; overground walking training: OWT; CON: control group.

**Table. S4D Node-splitting analysis of balance test batteries**

| comparison | k | prop | nma | direct | indir. | Diff | z | p-value |
| --- | --- | --- | --- | --- | --- | --- | --- | --- |
| BWS-TT vs CGT | 0 | 0 | 0.8366 | NA | 0.8366 | NA | NA | NA |
| BWS-TT vs CON | 4 | 0.69 | 0.4131 | 0.5062 | 0.2019 | 0.3043 | 0.7 | 0.4825 |
| BWS-TT vs OWT | 1 | 0.5 | 0.0399 | -0.0062 | 0.0864 | -0.0926 | -0.18 | 0.8554 |
| BWS-TT vs RA-GT | 0 | 0 | 0.2235 | NA | 0.2235 | NA | NA | NA |
| BWS-TT vs TC | 0 | 0 | 0.2305 | NA | 0.2305 | NA | NA | NA |
| BWS-TT vs TT | 1 | 0.23 | 0.1629 | -0.0895 | 0.24 | -0.3295 | -0.65 | 0.5189 |
| BWS-TT vs VR | 0 | 0 | 0.0065 | NA | 0.0065 | NA | NA | NA |
| CGT vs CON | 0 | 0 | -0.4235 | NA | -0.4235 | NA | NA | NA |
| CGT vs OWT | 0 | 0 | -0.7967 | NA | -0.7967 | NA | NA | NA |
| CGT vs RA-GT | 4 | 1 | -0.6132 | -0.6132 | NA | NA | NA | NA |
| CGT vs TC | 0 | 0 | -0.6061 | NA | -0.6061 | NA | NA | NA |
| CGT vs TT | 0 | 0 | -0.6737 | NA | -0.6737 | NA | NA | NA |
| CGT vs VR | 0 | 0 | -0.8301 | NA | -0.8301 | NA | NA | NA |
| OWT vs CON | 0 | 0 | 0.3732 | NA | 0.3732 | NA | NA | NA |
| RA-GT vs CON | 4 | 0.77 | 0.1896 | 0.0452 | 0.6741 | -0.6289 | -1.5 | 0.1334 |
| TC vs CON | 1 | 1 | 0.1826 | 0.1826 | NA | NA | NA | NA |
| TT vs CON | 4 | 0.57 | 0.2502 | 0.306 | 0.1766 | 0.1293 | 0.4 | 0.6907 |
| VR vs CON | 7 | 0.72 | 0.4066 | 0.4313 | 0.342 | 0.0893 | 0.26 | 0.7975 |
| OWT vs RA-GT | 0 | 0 | 0.1836 | NA | 0.1836 | NA | NA | NA |
| OWT vs TC | 0 | 0 | 0.1906 | NA | 0.1906 | NA | NA | NA |
| OWT vs TT | 3 | 0.74 | 0.123 | 0.0985 | 0.1911 | -0.0926 | -0.18 | 0.8554 |
| OWT vs VR | 0 | 0 | -0.0333 | NA | -0.0333 | NA | NA | NA |
| RA-GT vs TC | 0 | 0 | 0.0071 | NA | 0.0071 | NA | NA | NA |
| RA-GT vs TT | 1 | 0.17 | -0.0606 | 0.59 | -0.192 | 0.782 | 1.31 | 0.1906 |
| RA-GT vs VR | 1 | 0.24 | -0.2169 | 0.0544 | -0.3023 | 0.3566 | 0.7 | 0.4812 |
| TC vs TT | 0 | 0 | -0.0676 | NA | -0.0676 | NA | NA | NA |
| TC vs VR | 0 | 0 | -0.224 | NA | -0.224 | NA | NA | NA |
| TT vs VR | 2 | 0.37 | -0.1563 | -0.2249 | -0.1169 | -0.108 | -0.27 | 0.7903 |

Note: treadmill: TT; body-weight-supported treadmill training: BWS-TT; robot-assisted gait training: RA-GT; virtual-reality gait training: VR; conventional gait training: CGT; overground walking training: OWT; CON: control group.

**Table. S5A CINeMA confidence rating for dynamic steady-state balance**

| Comparison | Number of studies | Within-study bias | Reporting bias | Indirectness | Imprecision | Heterogeneity | Incoherence | Confidence rating |
| --- | --- | --- | --- | --- | --- | --- | --- | --- |
| BWS-TT:CGT | 1 | Some concerns | Low risk | No concerns | Major concerns | No concerns | No concerns | Low |
| BWS-TT:CON | 5 | Some concerns | Low risk | No concerns | No concerns | Major concerns | No concerns | Low |
| BWS-TT:OWT | 5 | Some concerns | Low risk | No concerns | Major concerns | No concerns | Major concerns | Very low |
| BWS-TT:RA-GT | 2 | Some concerns | Low risk | No concerns | Major concerns | No concerns | No concerns | Low |
| BWS-TT:TT | 4 | Some concerns | Low risk | No concerns | Major concerns | No concerns | No concerns | Low |
| CGT:RA-GT | 2 | Major concerns | Low risk | No concerns | Major concerns | No concerns | No concerns | Low |
| CGT:TT | 3 | No concerns | Low risk | No concerns | Major concerns | No concerns | No concerns | Moderate |
| CON:RA-GT | 4 | Major concerns | Low risk | No concerns | Major concerns | No concerns | No concerns | Low |
| CON:TT | 6 | No concerns | Low risk | No concerns | No concerns | Major concerns | No concerns | Moderate |
| CON:VR | 4 | Major concerns | Low risk | No concerns | Major concerns | No concerns | No concerns | Low |
| OWT:TT | 7 | Some concerns | Low risk | No concerns | Major concerns | No concerns | No concerns | Low |
| RA-GT:TT | 3 | Some concerns | Low risk | No concerns | Major concerns | No concerns | No concerns | Low |
| RA-GT:VR | 2 | Some concerns | Low risk | No concerns | Major concerns | No concerns | No concerns | Low |
| TT:VR | 1 | Some concerns | Low risk | No concerns | Major concerns | No concerns | No concerns | Low |
| BWS-TT:VR | 0 | Some concerns | Low risk | No concerns | Major concerns | No concerns | No concerns | Low |
| CGT:CON | 0 | No concerns | Low risk | No concerns | Major concerns | No concerns | No concerns | Moderate |
| CGT:OWT | 0 | Some concerns | Low risk | No concerns | Major concerns | No concerns | No concerns | Low |
| CGT:VR | 0 | No concerns | Low risk | No concerns | Major concerns | No concerns | No concerns | Moderate |
| CON:OWT | 0 | Some concerns | Low risk | No concerns | Major concerns | No concerns | No concerns | Low |
| OWT:RA-GT | 0 | Some concerns | Low risk | No concerns | Major concerns | No concerns | No concerns | Low |
| OWT:VR | 0 | Some concerns | Low risk | No concerns | Major concerns | No concerns | No concerns | Low |

Note: treadmill: TT; body-weight-supported treadmill training: BWS-TT; robot-assisted gait training: RA-GT; virtual-reality gait training: VR; conventional gait training: CGT; overground walking training: OWT; CON: control group.

**Table. S5B CINeMA confidence rating for static steady-state balance**

| Comparison | Number of studies | Within-study bias | Reporting bias | Indirectness | Imprecision | Heterogeneity | Incoherence | Confidence rating |
| --- | --- | --- | --- | --- | --- | --- | --- | --- |
| BWS-TT:CON | 1 | Some concerns | Low risk | No concerns | Major concerns | No concerns | Major concerns | Very low |
| CON:VR | 4 | Some concerns | Low risk | No concerns | Major concerns | No concerns | Major concerns | Very low |
| BWS-TT:VR | 0 | Some concerns | Low risk | No concerns | Major concerns | No concerns | Major concerns | Very low |

Note: treadmill: TT; body-weight-supported treadmill training: BWS-TT; robot-assisted gait training: RA-GT; virtual-reality gait training: VR; conventional gait training: CGT; overground walking training: OWT; CON: control group.

**Table. S5C CINeMA confidence rating for proactive balance**

| Comparison | Number of studies | Within-study bias | Reporting bias | Indirectness | Imprecision | Heterogeneity | Incoherence | Confidence rating |
| --- | --- | --- | --- | --- | --- | --- | --- | --- |
| BWS-TT:CON | 3 | No concerns | Low risk | No concerns | Major concerns | No concerns | No concerns | Moderate |
| BWS-TT:RA-GT | 2 | No concerns | Low risk | No concerns | Major concerns | No concerns | No concerns | Moderate |
| BWS-TT:TT | 1 | Some concerns | Low risk | No concerns | Major concerns | No concerns | No concerns | Low |
| CGT:RA-GT | 4 | Some concerns | Low risk | No concerns | Major concerns | No concerns | No concerns | Low |
| CGT:TT | 1 | No concerns | Low risk | No concerns | Major concerns | No concerns | No concerns | Moderate |
| CON:RA-GT | 1 | No concerns | Low risk | No concerns | Major concerns | No concerns | No concerns | Moderate |
| CON:TT | 2 | Some concerns | Low risk | No concerns | Major concerns | No concerns | No concerns | Low |
| CON:VR | 6 | Major concerns | Low risk | No concerns | Major concerns | No concerns | No concerns | Low |
| OWT:TT | 2 | Some concerns | Low risk | No concerns | Major concerns | No concerns | No concerns | Low |
| RA-GT:TT | 1 | Some concerns | Low risk | No concerns | Major concerns | No concerns | No concerns | Low |
| TT:VR | 3 | Some concerns | Low risk | No concerns | Major concerns | No concerns | No concerns | Low |
| BWS-TT:CGT | 0 | Some concerns | Low risk | No concerns | Major concerns | No concerns | No concerns | Low |
| BWS-TT:OWT | 0 | Some concerns | Low risk | No concerns | Major concerns | No concerns | No concerns | Low |
| BWS-TT:VR | 0 | No concerns | Low risk | No concerns | Major concerns | No concerns | No concerns | Moderate |
| CGT:CON | 0 | No concerns | Low risk | No concerns | Major concerns | No concerns | No concerns | Moderate |
| CGT:OWT | 0 | Some concerns | Low risk | No concerns | Major concerns | No concerns | No concerns | Low |
| CGT:VR | 0 | No concerns | Low risk | No concerns | Major concerns | No concerns | No concerns | Moderate |
| CON:OWT | 0 | Some concerns | Low risk | No concerns | Major concerns | No concerns | No concerns | Low |
| OWT:RA-GT | 0 | Some concerns | Low risk | No concerns | Major concerns | No concerns | No concerns | Low |
| OWT:VR | 0 | Some concerns | Low risk | No concerns | Major concerns | No concerns | No concerns | Low |
| RA-GT:VR | 0 | No concerns | Low risk | No concerns | Major concerns | No concerns | No concerns | Moderate |

Note: treadmill: TT; body-weight-supported treadmill training: BWS-TT; robot-assisted gait training: RA-GT; virtual-reality gait training: VR; conventional gait training: CGT; overground walking training: OWT; CON: control group.

**Table. S5D CINeMA confidence rating for balance test batteries**

| Comparison | Number of studies | Within-study bias | Reporting bias | Indirectness | Imprecision | Heterogeneity | Incoherence | Confidence rating |
| --- | --- | --- | --- | --- | --- | --- | --- | --- |
| BWS-TT:CON | 4 | No concerns | Low risk | No concerns | No concerns | Major concerns | No concerns | Moderate |
| BWS-TT:OWT | 1 | Major concerns | Low risk | No concerns | Major concerns | No concerns | No concerns | Low |
| BWS-TT:TT | 1 | No concerns | Low risk | No concerns | Major concerns | No concerns | No concerns | Moderate |
| CGT:RA-GT | 4 | Some concerns | Low risk | No concerns | No concerns | Major concerns | No concerns | Low |
| CON:RA-GT | 4 | Major concerns | Low risk | No concerns | Major concerns | No concerns | No concerns | Low |
| CON:TT | 4 | Some concerns | Low risk | No concerns | Major concerns | No concerns | No concerns | Low |
| CON:VR | 7 | Some concerns | Low risk | No concerns | No concerns | Major concerns | No concerns | Low |
| OWT:TT | 3 | Some concerns | Low risk | No concerns | Major concerns | No concerns | No concerns | Low |
| RA-GT:TT | 1 | Some concerns | Low risk | No concerns | Major concerns | No concerns | No concerns | Low |
| RA-GT:VR | 1 | Some concerns | Low risk | No concerns | Major concerns | No concerns | No concerns | Low |
| TT:VR | 2 | Some concerns | Low risk | No concerns | Major concerns | No concerns | No concerns | Low |
| BWS-TT:CGT | 0 | Some concerns | Low risk | No concerns | No concerns | Major concerns | No concerns | Low |
| BWS-TT:RA-GT | 0 | No concerns | Low risk | No concerns | Major concerns | No concerns | No concerns | Moderate |
| BWS-TT:VR | 0 | No concerns | Low risk | No concerns | Major concerns | No concerns | No concerns | Moderate |
| CGT:CON | 0 | Some concerns | Low risk | No concerns | Major concerns | No concerns | No concerns | Low |
| CGT:OWT | 0 | Some concerns | Low risk | No concerns | Major concerns | No concerns | No concerns | Low |
| CGT:TT | 0 | Some concerns | Low risk | No concerns | Major concerns | No concerns | No concerns | Low |
| CGT:VR | 0 | Some concerns | Low risk | No concerns | No concerns | Major concerns | No concerns | Low |
| CON:OWT | 0 | Some concerns | Low risk | No concerns | Major concerns | No concerns | No concerns | Low |
| OWT:RA-GT | 0 | Some concerns | Low risk | No concerns | Major concerns | No concerns | No concerns | Low |
| OWT:VR | 0 | Some concerns | Low risk | No concerns | Major concerns | No concerns | No concerns | Low |

Note: treadmill: TT; body-weight-supported treadmill training: BWS-TT; robot-assisted gait training: RA-GT; virtual-reality gait training: VR; conventional gait training: CGT; overground walking training: OWT; CON: control group.

**Table S6 PRISMA NMA Checklist of Items to Include When Reporting A Systematic Review Involving a Network Meta-analysis**

| **Section/Topic** | **Item #** | **Checklist Item** | **Reported on Page #** |
| --- | --- | --- | --- |
| **TITLE** |  |  |  |
| Title | 1 | Identify the report as a systematic review *incorporating a network meta-analysis (or related form of meta-analysis).* | Page 1 |
|  |  |  |  |
| **ABSTRACT** |  |  |  |
| Structured summary | 2 | Provide a structured summary including, as applicable:  **Background:** main objectives  **Methods:** data sources; study eligibility criteria, participants, and interventions; study appraisal; and *synthesis methods, such as network meta-analysis.*  **Results:** number of studies and participants identified; summary estimates with corresponding confidence/credible intervals; *treatment rankings may also be discussed. Authors may choose to summarize pairwise comparisons against a chosen treatment included in their analyses for brevity.*  **Discussion/Conclusions:** limitations; conclusions and implications of findings.  **Other:** primary source of funding; systematic review registration number with registry name. | Page 1 & 2 |
|  |  |  |  |
| **INTRODUCTION** |  |  |  |
| Rationale | 3 | Describe the rationale for the review in the context of what is already known*, including mention of why a network meta-analysis has been conducted.* | Page 2 & 3 |
| Objectives | 4 | Provide an explicit statement of questions being addressed, with reference to participants, interventions, comparisons, outcomes, and study design (PICOS). | Page 3 |
|  |  |  |  |
| **METHODS** |  |  |  |
| Protocol and registration | 5 | Indicate whether a review protocol exists and if and where it can be accessed (e.g., Web address); and, if available, provide registration information, including registration number. | Page 3 |
| Eligibility criteria | 6 | Specify study characteristics (e.g., PICOS, length of follow-up) and report characteristics (e.g., years considered, language, publication status) used as criteria for eligibility, giving rationale. *Clearly describe eligible treatments included in the treatment network, and note whether any have been clustered or merged into the same node (with justification).* | Page 3 & 4 |
| Information sources | 7 | Describe all information sources (e.g., databases with dates of coverage, contact with study authors to identify additional studies) in the search and date last searched. | Page 6 |
| Search | 8 | Present full electronic search strategy for at least one database, including any limits used, such that it could be repeated. | Page 3 & Table S1 |
| Study selection | 9 | State the process for selecting studies (i.e., screening, eligibility, included in systematic review, and, if applicable, included in the meta-analysis). | Page 4 |
| Data collection process | 10 | Describe method of data extraction from reports (e.g., piloted forms, independently, in duplicate) and any processes for obtaining and confirming data from investigators. | Page 4 |
| Data items | 11 | List and define all variables for which data were sought (e.g., PICOS, funding sources) and any assumptions and simplifications made. | Page 4 |
| **Geometry of the network** | **S1** | Describe methods used to explore the geometry of the treatment network under study and potential biases related to it. This should include how the evidence base has been graphically summarized for presentation, and what characteristics were compiled and used to describe the evidence base to readers. | Page 5 |
| Risk of bias within individual studies | 12 | Describe methods used for assessing risk of bias of individual studies (including specification of whether this was done at the study or outcome level), and how this information is to be used in any data synthesis. | Page 4 |
| Summary measures | 13 | State the principal summary measures (e.g., risk ratio, difference in means). *Also describe the use of additional summary measures assessed, such as treatment rankings and surface under the cumulative ranking curve (SUCRA) values, as well as modified approaches used to present summary findings from meta-analyses.* | Page 4 |
| Planned methods of analysis | 14 | Describe the methods of handling data and combining results of studies for each network meta-analysis. This should include, but not be limited to:   - *Handling of multi-arm trials;* - *Selection of variance structure;* - *Selection of prior distributions in Bayesian analyses; and* - *Assessment of model fit.* | Page 5 |
| **Assessment of Inconsistency** | **S2** | Describe the statistical methods used to evaluate the agreement of direct and indirect evidence in the treatment network(s) studied. Describe efforts taken to address its presence when found. | Page 4 |
| Risk of bias across studies | 15 | Specify any assessment of risk of bias that may affect the cumulative evidence (e.g., publication bias, selective reporting within studies). | Page 4 |
| Additional analyses | 16 | Describe methods of additional analyses if done, indicating which were pre-specified. This may include, but not be limited to, the following:   - Sensitivity or subgroup analyses; - Meta-regression analyses; - *Alternative formulations of the treatment network; and* - *Use of alternative prior distributions for Bayesian analyses (if applicable).* | Page 5 |
|  |  |  |  |
| **RESULTS†** |  |  |  |
| Study selection | 17 | Give numbers of studies screened, assessed for eligibility, and included in the review, with reasons for exclusions at each stage, ideally with a flow diagram. | Figure 1 |
| **Presentation of network structure** | **S3** | Provide a network graph of the included studies to enable visualization of the geometry of the treatment network. | Figure 3A & 3B &  3C & 3D |
| **Summary of network geometry** | **S4** | Provide a brief overview of characteristics of the treatment network. This may include commentary on the abundance of trials and randomized patients for the different interventions and pairwise comparisons in the network, gaps of evidence in the treatment network, and potential biases reflected by the network structure. | Page 6 & 7 |
| Study characteristics | 18 | For each study, present characteristics for which data were extracted (e.g., study size, PICOS, follow-up period) and provide the citations. | Table S2 |
| Risk of bias within studies | 19 | Present data on risk of bias of each study and, if available, any outcome level assessment. | Page 6 |
| Results of individual studies | 20 | For all outcomes considered (benefits or harms), present, for each study: 1) simple summary data for each intervention group, and 2) effect estimates and confidence intervals. *Modified approaches may be needed to deal with information from larger networks.* | Table S2 |
| Synthesis of results | 21 | Present results of each meta-analysis done, including confidence/credible intervals. *In larger networks, authors may focus on comparisons versus a particular comparator (e.g. placebo or standard care), with full findings presented in an appendix. League tables and forest plots may be considered to summarize pairwise comparisons.* If additional summary measures were explored (such as treatment rankings), these should also be presented. | Figure 4A & 4B &  4C & 4D |
| **Exploration for inconsistency** | **S5** | Describe results from investigations of inconsistency. This may include such information as measures of model fit to compare consistency and inconsistency models, *P* values from statistical tests, or summary of inconsistency estimates from different parts of the treatment network. | Page 6 & 7 |
| Risk of bias across studies | 22 | Present results of any assessment of risk of bias across studies for the evidence base being studied. | Page 6 |
| Results of additional analyses | 23 | Give results of additional analyses, if done (e.g., sensitivity or subgroup analyses, meta-regression analyses*, alternative network geometries studied, alternative choice of prior distributions for Bayesian analyses,* and so forth). | Page 6 & 7 & 8 |
|  |  |  |  |
| **DISCUSSION** |  |  |  |
| Summary of evidence | 24 | Summarize the main findings, including the strength of evidence for each main outcome; consider their relevance to key groups (e.g., healthcare providers, users, and policy-makers). | Page 8 & 9 |
| Limitations | 25 | Discuss limitations at study and outcome level (e.g., risk of bias), and at review level (e.g., incomplete retrieval of identified research, reporting bias). *Comment on the validity of the assumptions, such as transitivity and consistency. Comment on any concerns regarding network geometry (e.g., avoidance of certain comparisons).* | Page 9 |
| Conclusions | 26 | Provide a general interpretation of the results in the context of other evidence, and implications for future research. | Page 9 |
|  |  |  |  |
| **FUNDING** |  |  |  |
| Funding | 27 | Describe sources of funding for the systematic review and other support (e.g., supply of data); role of funders for the systematic review. This should also include information regarding whether funding has been received from manufacturers of treatments in the network and/or whether some of the authors are content experts with professional conflicts of interest that could affect use of treatments in the network. | Page 10 |
